# Kidney Injury in COVID-19: Epidemiology, Molecular Mechanisms and Potential Therapeutic Targets

**DOI:** 10.3390/ijms23042242

**Published:** 2022-02-17

**Authors:** J. Pedro Teixeira, Sharon Barone, Kamyar Zahedi, Manoocher Soleimani

**Affiliations:** 1 Department of Internal Medicine, Division of Nephrology, University of New Mexico Health Sciences Center, Albuquerque, NM 87131, USA; sbarone@salud.unm.edu (S.B.); kzahedi@salud.unm.edu (K.Z.); 2 Department of Internal Medicine, Division of Pulmonary, Critical Care, and Sleep Medicine, University of New Mexico Health Sciences Center, Albuquerque, NM 87131, USA; 3 Research/Medicine Services, New Mexico Veterans Healthcare Medical Center, Albuquerque, NM 87108, USA

**Keywords:** SARS-CoV-2, COVID-19, acute kidney injury

## Abstract

As of December 2021, SARS-CoV-2 had caused over 250 million infections and 5 million deaths worldwide. Furthermore, despite the development of highly effective vaccines, novel variants of SARS-CoV-2 continue to sustain the pandemic, and the search for effective therapies for COVID-19 remains as urgent as ever. Though the primary manifestation of COVID-19 is pneumonia, the disease can affect multiple organs, including the kidneys, with acute kidney injury (AKI) being among the most common extrapulmonary manifestations of severe COVID-19. In this article, we start by reflecting on the epidemiology of kidney disease in COVID-19, which overwhelmingly demonstrates that AKI is common in COVID-19 and is strongly associated with poor outcomes. We also present emerging data showing that COVID-19 may result in long-term renal impairment and delve into the ongoing debate about whether AKI in COVID-19 is mediated by direct viral injury. Next, we focus on the molecular pathogenesis of SARS-CoV-2 infection by both reviewing previously published data and presenting some novel data on the mechanisms of cellular viral entry. Finally, we relate these molecular mechanisms to a series of therapies currently under investigation and propose additional novel therapeutic targets for COVID-19.

## 1. Introduction

The major focus of healthcare since the identification of the novel coronavirus SARS-CoV-2 in December 2019 has been on the prevention of infection and treatment of patients who have contracted the virus. As of December 2021, SARS-CoV-2 has infected over 250 million people worldwide, resulting in over five million deaths [1]. Though its primary manifestation is respiratory disease, COVID-19, especially when severe, can induce dysfunction or failure of multiple organs, including the kidneys [2]. Indeed, it has been claimed that the kidney is the second most common organ affected by COVID-19 after the lungs [3]. This review will focus on the epidemiology, clinical features, and pathogenesis of kidney injury in SARS-CoV-2 infection and its long-lasting effects on kidney function.

## 2. Epidemiology and Clinical Impact of Kidney Injury in COVID-19

### 2.1. Epidemiology of AKI in COVID-19

Acute kidney injury (AKI) and particularly AKI requiring renal replacement therapy (RRT) have previously been associated with increased mortality across a variety of settings, but renal dysfunction may carry particular prognostic significance in the setting of COVID-19 [4,5]. The rates of AKI complicating COVID-19 have varied throughout the pandemic. Early published reports of hospitalized patients with COVID-19 from China reported relatively low rates of AKI, at 10% or less [6,7,8,9,10]. However, subsequent reports of Chinese cohorts revealed substantially higher rates of kidney involvement, particularly among critically ill patients [11,12,13,14]. In contrast, the rates of AKI among patients hospitalized during the first COVID-19 wave in the United States were substantially higher than those reported from China [15,16,17]. More recently, studies from the US have report AKI rates ranging from 32 to 57% overall, with 9–20% developing AKI requiring RRT (AKI-RRT) [18,19,20]. In one of these US studies, 3345 COVID-19 patients were compared to nearly 10,000 COVID-19-negative historical controls. COVID-19-positive patients developed AKI at twice the rate (57% vs. 25%), suggesting COVID-19 specifically predisposes patients to AKI more than other forms of acute illness. The COVID-19 patients also had an increased need for RRT, ICU admission, and mechanical ventilation and experienced higher in-hospital mortality [19]. Published rates of AKI in European cohorts have been similarly high, with one retrospective cohort of 4700 patients found to have an AKI rate of 26% [21]. Smaller European cohorts of ICU patients with COVID-19 have been reported with AKI rates ranging between 50 and 80% [22,23,24,25]. Some analyses have suggested that AKI rates have decreased through subsequent waves of the pandemic; although the reasons for this are unclear, it has been speculated that AKI rates have decreased as patient demographics have changed and management of COVID-19 has improved [20,26,27,28]. Nonetheless, in even these more recent COVID-19 cohorts, 15–30% of hospitalized patients develop AKI [20,26]. A recent international meta-analysis found a pooled prevalence of AKI in 28% of hospitalized patients, a rate that rose to 45% when considering only ICU patients, with AKI-RRT rates of 9% among all hospitalized patients and 19% among ICU patients [29]. Notably, AKI appears to disproportionately affect racial and ethnic minorities [15,19,20,30].

### 2.2. Mortality of COVID-19-Associated AKI

Despite significant geographic variability in AKI risk, the impact on outcomes has been consistent, with AKI and other renal abnormalities consistently linked with an increased risk of death [14,20]. In a prospective cohort of 701 patients admitted with COVID-19 in China, the rate of AKI was only 5.1%, with proteinuria seen in 44% and hematuria in 27% of patients [7]. In multivariable analysis, moderate-to-severe AKI was found in 1.3 and 1.2% (stages 2 and 3, respectively) of COVID-19 patients and associated with increased mortality [7]. Likewise, proteinuria and hematuria were independently associated with an increased risk of death, with hazard ratios ranging from 2.5 for low-grade proteinuria to 8.9 for high-grade hematuria [7]. Similarly, in a retrospective cohort of 333 Chinese patients admitted with COVID-19 pneumonia, over 75% presented with some type of renal abnormality, which was associated with a nearly 10-fold increased mortality rate (11.2% vs. 1.2%) [31]. The prognostic significance of hematuria and proteinuria was replicated in a US cohort in which proteinuria or hematuria was associated with AKI development, need for ICU admission or mechanical ventilation, and death [32]. Even studies with a low overall incidence of AKI found it to be independently associated with an increased risk of death [25,33,34]. As expected, outcomes are worst in those who develop severe AKI, especially AKI-RRT. In US cohorts, AKI-RRT develops in 20–45% of critically ill COVID-19 patients, with AKI-RRT in COVID-19 consistently associated with an increased risk of death and carrying a short-term mortality rate of ≥50% across diverse cohorts, a finding that we replicated in a cohort of the first 30 COVID-19 patients with AKI treated locally with continuous renal replacement therapy (CRRT) [15,16,17,18,19,35,36,37]. A recent multinational cross-sectional observational study from 168 hospitals in 16 countries comprising over 20,600 patients admitted with COVID-19 reported an overall hospital mortality rate of 19%. However, the mortality in the cohort increased in association with higher levels of organ support (namely mechanical ventilation, vasopressors, or new RRT), ranging from 9% in patients who did not require organ support to >70% in patients requiring all three forms of organ support [38].

### 2.3. Relationship between COVID-19, Pre-Existing CKD, and Risk of Future CKD

Patients presenting with pre-existing kidney conditions, such as chronic kidney disease (CKD), including both end-stage kidney disease (ESKD) and pre-dialysis CKD, are also at greater risk for poor outcomes from COVID-19. Baseline kidney disease has been consistently found to be an independent risk factor for severe disease or mortality from COVID-19, even when adjusting for confounding risk factors, such as underlying diabetes, hypertension, and cardiovascular disease [18,39,40,41,42,43,44,45,46,47,48,49,50,51,52,53,54]. Despite the clear connection between baseline kidney function and COVID-19 severity, far fewer data have been published to elucidate the effect of survival from COVID-19 on subsequent long-term kidney function, but four recent analyses of large datasets suggest a link [55,56,57,58]. In particular, data are emerging suggesting that “long COVID”, the clinical syndrome of post-acute sequelae involving pulmonary and extrapulmonary organ systems, may significantly affect the kidneys [55].

In a cohort study of over 1700 admissions for COVID-19 in China, 35% of patients had reduced kidney function, defined as estimated glomerular filtration rate (eGFR) < 90 mL/min/1.73 m^2^, six months after discharge, notably including 13% who did not have AKI during the index admission [57]. Similarly, a retrospective-propensity-score analysis of over 27,000 COVID-19 patients in the US found the hazard ratio for both AKI and CKD development to be higher than that of general-population controls or controls with other viral lower respiratory infections [58].

The largest and most comprehensive analysis to date of the long-term renal effects of COVID-19 is a cohort study from the US Veterans Health Administration. The study examined nearly 90,000 30-day COVID-19 survivors and determined that they had a higher risk of adverse kidney outcomes, including AKI, eGFR decline, and major adverse kidney events (MAKE, a composite of decline in eGFR ≥ 50%, ESKD, or death) compared to control patients [55]. Notably, though the association with decreased kidney function was strongest in more severe COVID-19 cases, the relationship was attenuated but persisted in patients that did not require hospitalization or develop AKI [55,56].

Finally, a fourth study supports the idea that COVID-19 may somehow predispose to worsening long-term kidney function independently of AKI diagnosed during the index COVID-19 admission. A retrospective analysis of 1612 patients with AKI, including 182 with COVID-19-associated AKI and 1430 without COVID-19 admitted to five US hospitals, found, after adjusting for comorbidities and severity of AKI, that eGFR declined by over 11 mL/min/1.73 m^2^ per year faster in the COVID-19-associated AKI patients than in the COVID-19-negative AKI patients [59].

Taken together, these data suggest that COVID-19 may predispose patients to CKD independently of clinically apparent AKI. Though the mechanism for such an effect remains to be elucidated, a preliminary study of 23 COVID-19-positive inpatients suggests that subclinical AKI may be common in patients with COVID-19, as determined by elevated levels of the product of urinary biomarker tissue inhibitors of metalloproteinases-2 and insulin-like growth factor binding protein 7 ([TIMP-2] • [IGFBP7]) without significant change in serum creatinine [24].

## 3. Clinical and Histopathological Features of Kidney Injury in COVID-19

### 3.1. Tubular Injury and Tubular Dysfunction as Primary Features of COVID-19-Associated AKI

Multiple case series, including autopsy and biopsy studies, have shown that kidney disease in the setting of COVID-19 is most commonly due to acute tubular injury (ATI), often somewhat less severe than would be expected for the clinical severity of AKI; however, multiple glomerular pathologies, especially collapsing glomerulopathy, have also been reported [17,60,61,62,63,64,65,66,67,68,69,70,71,72,73,74]. Collapsing glomerulopathy appears to occur most commonly in patients expressing high-risk APOL1 alleles [65,66,67,68,69,70,71]. Thrombotic findings, including arterial thrombi, arteritis, and glomerular microthrombi, have been reported to a lesser degree [69,70,71,72,73,74,75,76].

ATI as the predominant cause of AKI in COVID-19 has been confirmed in additional observational studies in which the cause of AKI is determined, rather than by histopathology, on clinical grounds combined with urine microscopy [17,77]. ATI as the primary lesion of COVID-19-associated AKI is also consistent with a urinary-biomarker study demonstrating that COVID-19 patients with stage 2 and 3 AKI have elevated levels of urinary [TIMP-2] • [IGFBP7] and tubular (α-1-microglobulin) proteinuria [24,78]. In another study of 49 COVID-19 patients hospitalized in Belgium, investigators found that most patients had evidence of specific dysfunction of the proximal tubules, with low-molecular-weight proteinuria in 70–80%, aminoaciduria in 46%, abnormal uricosuria in 46%, and abnormal phosphaturia in 19%, with abnormal uricosuria associated with increased disease severity [79]. In six patients that underwent autopsy, the investigators found prominent ATI of the initial segment of the proximal tubule, with disruption of the brush border. Another report of 42 patients with COVID-19 in France similarly documented the presence of incomplete Fanconi syndrome in the majority of patients and noted that tubular dysfunction often precedes clinically overt AKI [80].

More recent data suggest that, though not entirely overlapping, ATI in COVID-19 may share similar molecular mechanisms as septic AKI. A recent multi-omics study of seven COVID-19 kidney autopsy specimens demonstrated a similar transcriptomic and proteomic profile to that of seven control samples with non-COVID-19 septic AKI, with both profiles prominently featuring the presence of mitochondrial dysfunction [62].

### 3.2. Hemoproteinuria in COVID-19-Associated AKI

Despite tubular injury being the predominant pathologic lesion, proteinuria and hematuria appear to be relatively prominent in COVID-19-associated AKI [7,15,17,24,31,32]. Specifically, >40% of patients hospitalized for COVID-19 have proteinuria when assessed by qualitative urinalysis [7,15,32,81,82]. Similarly high rates of proteinuria were observed in two studies in which patients admitted with COVID-19 underwent quantitative proteinuria assessment, with 19 (83%) of 23 patients (of which 12 developed AKI) having proteinuria >150 mg per g creatinine in one study and 33 (63.5%) of 52 patients (of which 34 developed AKI) having albuminuria of >30 mg per g creatinine in the other [24,83].

### 3.3. Time Course of COVID-19-Associated AKI

Though several studies suggest that most AKI in patients hospitalized with COVID-19 is diagnosed within the first day of admission [15,16,20], hospital-acquired AKI appears to carry a significantly worse prognosis [30], likely representing worsening multiorgan dysfunction and development of critical illness associated with excess systemic inflammation [64,84,85]. Likewise, AKI in COVID-19 appears to be closely tied to the development and progression of respiratory failure. In a large cohort of over 5000 patients from New York, compared to 22% of non-ventilated patients, 90% of patients on mechanical ventilation developed AKI, with approximately 50% developing AKI within 24 h of initiation of mechanical ventilation [15]. It is unclear whether this temporal relationship is due to the effects of invasive mechanical ventilation itself or due to the overall progression of COVID-19, leading to multiorgan dysfunction simultaneously affecting the kidney and lungs.

## 4. Pathogenesis of Kidney Injury in COVID-19: Indirect vs. Direct Kidney Injury

### 4.1. Indirect Contributors to COVID-19-Associated AKI

It has been proposed that kidney disease in COVID-19 is a result of both indirect and direct effects of SARS-CoV-2 infection [2,86]. Many cases appear to be clearly attributable to indirect effects, such as ischemic injury from hypotension and hypoxemia, non-COVID-19 sepsis, and/or toxic injury from nephrotoxins or rhabdomyolysis [17,84,87].

Interestingly, some studies suggest that even indirect contributors to AKI may be somewhat specifically enhanced in patients with COVID-19. For example, mechanical ventilation is known, in part via decreased renal blood flow and potentially the hemodynamic effects of intravenous sedation, to be associated with an increased risk of AKI [88,89,90,91]. However, in a prospective pilot study of 30 mechanically ventilated patients (15 with COVID-19 and 15 with ARDS due to other causes), the COVID-19 patients had decreased renal blood flow despite similar ventilatory management [92]. Similarly, in an analysis of over 750 patients hospitalized with influenza or COVID-19, the risk of stage 3 AKI was higher in the COVID-19 cohort than the influenza group, and mechanical ventilation was associated with an increased risk of AKI in the COVID-19 group but not the influenza cohort [93].

Other than toxic or ischemic injury, additional indirect causes of AKI that have been proposed include cytokine storm, activation and dysregulation of the angiotensin II pathway and complement system, endothelial dysfunction, abnormal platelet activation, hypercoagulation, and microangiopathy [2,3,28,94,95,96,97]. The direct contribution of viral infection to AKI in the setting of COVID-19 has been highly debated [28,86,96,97,98,99,100,101,102,103,104,105,106,107,108]. For example, in one cohort study of over 500 patients admitted with COVID-19, 161 (28%) developed AKI, and a detailed clinical analysis was carried out to determine the cause of AKI. The bulk of the 161 AKI patients were felt to have suffered ischemic or toxic renal insults, but in 13% of cases, the investigators found no potential cause of AKI other than possible direct SARS-CoV-2-mediated renal damage [17].

### 4.2. COVID-19 as a Direct Cause of AKI: Tissue Studies

The presence of AKI with no overt hemodynamic or toxic origin in 13% of the patients in the abovementioned study raises the possibility that the virus can directly affect the kidney. The plausibility of direct infection of the kidney by SARS-CoV-2 stems, in part, from the fact that the receptors for cellular viral entry, including angiotensin-converting enzyme-2 (ACE2), are highly expressed in the proximal tubule, particularly in the apical brush border of the proximal tubule [109,110,111,112,113,114]. ACE2 is also expressed, to a lesser degree, by glomerular parietal cells/podocytes, which could potentially be related to the significant proteinuria seen in COVID-19-associated AKI, although preliminary analyses suggest this proteinuria is primarily tubular in origin [24,110,111]. Noteworthy is the fact that the localization of ACE2 receptors in the kidney matches the regions of damage found in COVID-19 patients with AKI.

Many of the initial studies arguing for a direct viral effect in COVID-19-associated AKI included reports of virus-like particles detected in the renal epithelium of autopsy and biopsy samples by electron microscopy (EM) [60,79,98,115,116,117,118]. However, multiple publications have questioned the specificity of EM findings for SARS-CoV-2 detection, as the virus-like particles appear similar to normal endocytic vesicles or multivesicular bodies, and particles with a similar appearance have been found in COVID-19-negative patients [101,102,103,104,105]. As result, subsequent studies on the possible direct effect of viral infection in COVID-19-associated AKI have shifted towards the attempted detection of viral protein, RNA, or intact virus in the renal epithelium.

The first published study to demonstrate the presence of SARS-CoV-2 protein in kidney tissue was an autopsy series by Su et al. of 26 patients who died of COVID-19 [60]. Of these patients, nine developed AKI and/or new-onset proteinuria, and five required CRRT. In addition to reporting virus-like particles on EM, the authors were able to detect SARS-CoV-2 nucleoprotein by immunofluorescence (IF) in the tubular epithelium in three of six patients with AKI [60]. In another study of autopsy samples from COVID-19 patients, analysis of viral load in multiple tissue types demonstrated that the kidneys had high detectable levels of viral RNA copies per cell. Viral spike protein was also detected by indirect IF in multiple kidney compartments (i.e., glomeruli, endothelium, and tubules) [119].

In a recently published manuscript, Diao et al. described a cohort of 85 patients admitted with COVID-19 with a 27% rate of AKI [106]. Post-mortem analyses of the patients with moderate or severe acute tubular injury demonstrated the accumulation of SARS-CoV-2 viral protein in kidney tubules by immunohistochemistry (IHC) and viral RNA by in situ hybridization (ISH). The presence of viral proteins (e.g., nucleocapsid and spike proteins) was restricted to tubules that were also ACE2-positive by IHC. The presence of the virus was associated with tubulointerstitial infiltration of macrophages and deposition of the complement membrane attack complex (C5b-C9), implicating these components of the inflammatory and immune systems as possible mediators of tubular damage resulting from viral infection.

A study by Braun et al. demonstrated that infective virus can be isolated from kidney tissue [120]. In these studies, non-transfected cultured Vero E6 cells were incubated with kidney homogenates from SARS-CoV-2 PCR-positive and PCR-negative samples [120]. The authors found that cells incubated with PCR-positive homogenates had significantly increased (1000-fold) viral mRNA compared to cultured cells exposed to PCR-negative homogenates [120].

However, not all attempts to detect SARS-CoV-2 protein or RNA in the kidneys of pathologic specimens have been successful [64,108,121,122,123]. In one such study, IHC and ISH examination of 10 kidney biopsy samples failed to detect SARS-CoV-2 nucleoproteins or RNA [123]. In another, the investigators also failed to detect viral RNA by single-nucleus RNA sequencing in autopsy samples [124]. Furthermore, another autopsy study, which failed to demonstrate significant inflammatory infiltration of the kidneys, despite demonstration of viral RNA in kidney tissue and spike protein in renal tubular epithelium, suggested that the presence of virus does not always correlate with kidney injury [125]. Delorey et al. used sn-RNAseq to detect the expression of SARS-CoV-2 in the heart, kidney, and lungs. The replication of virus was entirely cytoplasmic and never occurred anywhere near the nucleus. If sc-RNAseq rather than sn-RNAseq had been performed, the results may have been different.

### 4.3. COVID-19 as a Direct Cause of AKI: Detection of Virus and Viral Particles in Urine

In addition to attempting to isolate virus or viral particles in kidney tissue, multiple studies have attempted to detect SARS-CoV-2 in urine, with highly variable results. While multiple studies have reported the presence of detectable viral RNA in the urine of COVID-19 patients, many others have failed to do so [8,9,10,107,126,127,128,129,130,131,132,133,134,135,136,137,138,139]. Two systemic reviews suggest that the rate of PCR positivity for SARS-CoV-2 of urine from COVID-19-infected patients is 0–4% [140,141]. Although the overall rates of PCR positivity of urine appear to be low, interestingly, urine or stool samples from COVID-19 patients may remain PCR-positive even after respiratory symptoms have resolved and nasopharyngeal PCR has turned negative [130,132].

The presence of virus in the urine not only suggests the possibility of a direct viral effect upon the kidney, but a recent study implies that SARS-CoV-2 viruria may have additional important clinical implications [83]. The analysis of spun urinary sediments of patients with PCR-confirmed COVID-19 using qRT-PCR demonstrated that the average viral load was four times higher in COVID-19 patients with AKI than those who did not develop AKI. Further analysis of the results suggested that viral load strongly correlated with mortality but not with AKI stage or albuminuria [83]. These results are in contrast with another observational study in which SARS-CoV-2 RNA levels were measured by qRT-PCR in the urine of 81 patients with COVID-19 admitted to the ICU [107]. Despite a high rate of AKI (63%), urinary PCR was positive in only 7% (*n* = 6) of the overall cohort and only 10% (*n* = 5) of the AKI patients. Detection of viral RNA was not associated with development of renal dysfunction, need for RRT, overall severity of illness, or mortality [107]. The discrepant results of these studies may be due to the use of differentially processed samples (e.g., urinary sediment, which may be enriched with viral RNA, versus whole urine) and the use of different qRT-PCR methodologies [83].

### 4.4. COVID-19 as a Direct Cause of AKI: Experiments Using Kidney Spheroids and Organoids

Additional studies of the possible viral effects on kidney tissue have been carried out using kidney organoids or spheroids. Organoids and spheroids, with organoids being somewhat more complex and larger, are 3D multicellular in vitro tissue constructs designed to mimic the corresponding in vivo organs or tissues, such as renal tubular epithelium [142,143,144]. To test whether SARS-CoV-2 can infect kidney tubular cells, Monteil et al. generated kidney organoids from pluripotent stem cells [145]. These organoids expressed podocyte and proximal tubular cell markers, as well as ACE2, and could be infected by SARS-CoV-2 [145]. Soluble ACE2 blocked SARS-CoV-2 infection in a dose-dependent manner, confirming that ACE2 is the SARS-CoV-2 binding entity in proximal tubule cells and podocytes [145]. Omer et al. took these results a step further by generating human kidney monolayers and spheroids, grafting the latter into mice by subcutaneous injection to generate tubular structures, and demonstrated that the monolayers and spheroids express ACE2 and the co-receptor for SARS-CoV-2 viral entry, transmembrane protease serine 2 (TMPRSS2) [84,146]. They subsequently demonstrated that SARS-CoV-2 can infect the monolayers and spheroids by measuring viral load in the cells by qRT-PCR and performing IF microscopy [84]. However, notably, unlike a control Vero E6 cell line, the monolayers and spheroids did not demonstrate any cytopathic effect when examined by light microscopy [84]. Furthermore, though the more rapidly proliferating monolayers did demonstrate changes in gene expression typical of AKI, including fibrosis-related genes and those involved in dedifferentiation and epithelial–mesenchymal transition, such changes were absent in the more slowly replicating spheroids [84]. In contrast, unlike the Vero E6 cells, the spheroids and monolayer cells expressed genes associated with activation of the type 1 interferon response, which may have conferred resistance to viral cytopathic damage [84]. Omer et al. argue that their results suggest that non-human Vero cells, despite extensive use in prior coronavirus studies, may not be the best cell model in which to study the renal effects of SARS-CoV-2 [83,84,120,127,147]. They also suggest that the differences in AKI-related gene activation in the monolayers and the spheroids imply that direct viral infection may not cause significant tubular damage in uninjured quiescent renal tissue but, once AKI-induced cell proliferation is triggered by non-viral kidney injury, viral infection and replication aggravate the damage.

## 5. Pathogenesis of Kidney Injury in COVID-19: Molecular Mechanisms and Potential Molecular Targets

### 5.1. Interaction of SARS-CoV-2 with the Cell Membrane

**Figure 1:**
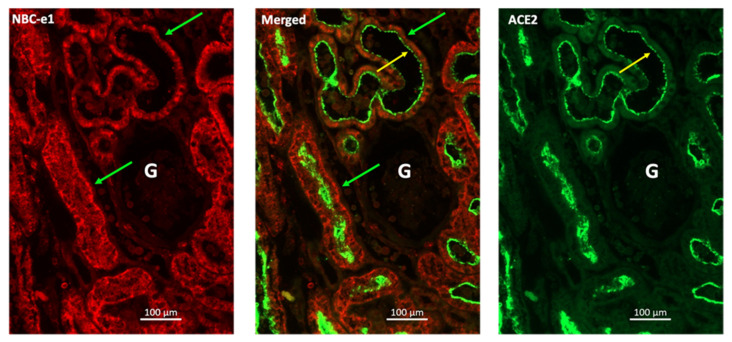
Immunofluorescence labeling of ACE2 in human kidney sections. Double-label immunofluorescence images in human kidney using antibodies against ACE2 and NBCe1. As indicated, the images depict the apical labeling of ACE2 (**right panel**; green) and basolateral labeling of NBC-e1 (**left panel**; red) in proximal tubule cells. Merged image is present in the middle panel. Green arrows indicate basolateral NBC-e1 staining and yellow arrows denote apical ACE2 staining. G represents glomerulus. (*Unpublished data from the author’s laboratory*).

**Figure 2:**
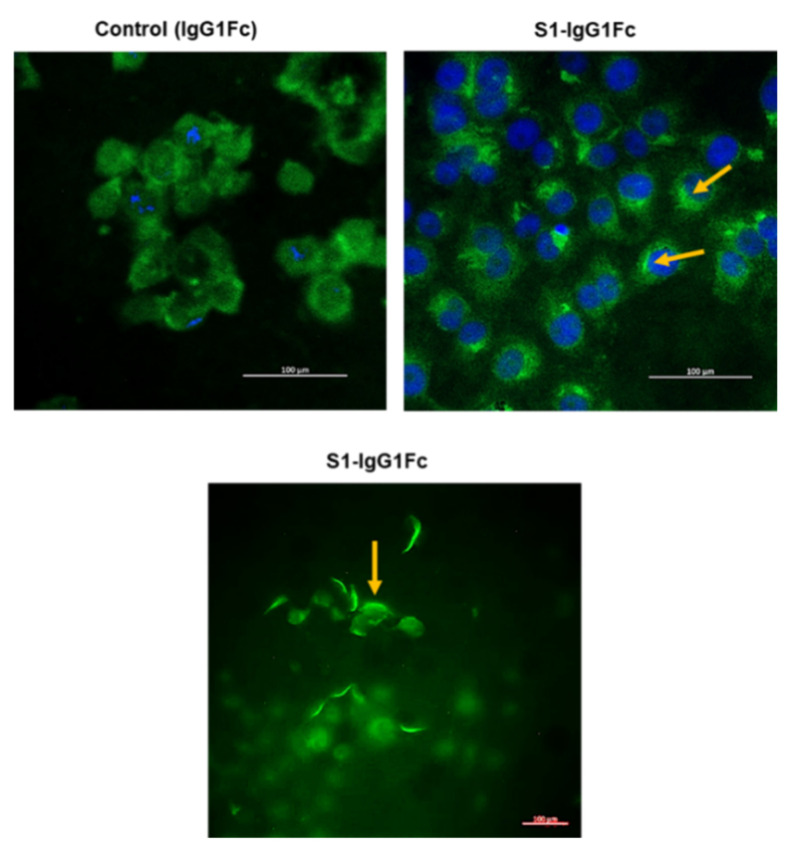
Binding of S1-spike-conjugated IgG1 Fc fragment to Vero E6 cells. Spike protein, specifically the S1 component, mediates the binding of SARS-CoV-2 to the cell membrane via angiotensin-converting enzyme 2 (ACE II). Binding of S1 Fc-GFP and Fc-GFP (with no S1 component) to Vero E6 cells, as visualized by a Zeiss Airyscan microscope (Section 7.2), shows significant and specific binding of S1 Fc-GFP beads to Vero E6 cells. The top panels were performed in the presence of DAPI nuclear stain. The bottom panel depicts the binding of spike protein (S1 Fc-GFP) to Vero 6 cells in the absence of DAPI nuclear stain. These studies clearly indicate that the SARS-CoV-2 spike protein can directly bind with the kidney epithelial Vero 6 cells. The orange arrows indicate specific S1 Fc-GFP signal. (*Unpublished data from the author’s laboratory*).

Cellular infection by SARS-CoV-2 is a multi-step process [148]. The virus initiates the process of cellular entry by binding to the ACE2 receptor via the S1 region of its spike protein. Upon binding to ACE2, SARS-CoV-2 spike protein undergoes proteolytic processing by furin and TMPRSS2, a process that is necessary for the virus to enter the cell [146,149]. This process of viral entry has been the subject of intense research, including in our own lab, where we have analyzed this process in human kidney tissue and cultured kidney epithelial cells. Using IF microscopy, we examined the presence of ACE2 on the apical membrane of human kidney proximal tubule cells (Figure 1). These studies were performed using antibodies against ACE2 and the basolateral Na^+^-HCO_3_^−^ cotransporter NBCe1. As indicated, ACE2 shows distinct localization on the apical membrane of kidney proximal tubule cells (Figure 1). These data are consistent with the published literature and support the notion that human kidney proximal tubule cells can potentially bind with SARS-CoV-2. Next, we examined the binding of GFP-tagged S1 protein (S1-IgG1Fc-GFP) to the cell surface of Vero E6 cells. Vero 6 cells, kidney epithelial cells isolated from African green monkey, display many properties of human kidney proximal tubule cells and are used extensively in laboratory experiments of coronavirus infection and replication [147]. The results were compared to those of IgG1Fc protein adsorbed to protein-G-coated fluorescent beads (IgG1Fc-GFP) not bound to S1 protein in order to determine the non-specific background binding. Comparison of fluorescence staining of the top left panel (non-specific binding) vs. the top right and bottom panels (S1-mediated binding), as shown in Figure 2, indicated significant binding of S1-IgG1Fc-GFP to Vero-E6 cells vs. IgG1Fc-GFP. These results strongly support the binding of SARS-CoV-2 to the kidney proximal tubule cell membrane.

### 5.2. Potential Therapeutic Targets: An Overview

The steps of virus–cell membrane fusion, entry via endocytosis and processing within endosomal vesicles, and RNA replication by RNA-dependent RNA polymerase (RdRP) have all been investigated as potential targets of antiviral therapies. For example, vaccines against the spike protein can lead to the generation of antibodies that prevent the virus from binding to its target. Interference with preprocessing of the spike protein by furin has been shown, in preclinical investigations, to reduce viral infectivity and syncytia formation and may be an effective therapeutic approach [149,150,151]. Likewise, inhibition of TMPRSS2 may potentially be used to prevent the processing of the spike protein and thwart cellular entry by the virus [146]. As outlined below, interfering with the acidification of endosomes may potentially avert viral uncoating and the release of its RNA genome into the cytosol. Finally, inhibition of viral RdRP by agents such as remdesivir blocks the synthesis of viral RNA needed for virion formation [152,153,154].

### 5.3. Potential Therapeutic Targets: Lysosomal Acidification

As previously alluded to, a key step in cellular infection by SARS-CoV-2 and other coronaviruses is lysosomal acidification, which is essential for the pH-dependent cleavage of viral glycoproteins by endosomal proteases and, ultimately, viral replication [155,156]. Three categories of pharmacologic agents can potentially inhibit endosomal/lysosomal acidification [156]. The first class of agents is comprised of weak bases, such as chloroquine, ammonium chloride, and amantadine, which diffuse across endosomal/lysosome membranes and become protonated, thereby releasing base-equivalent OH^−^ and alkalinizing the endosomal/lysosomal internal environment. The second class of agents comprises inhibitors of vacuolar H^+^-ATPases (e.g., bafilomycin A1 and concanamycin A), which have been successfully used to prevent endosomal/lysosomal acidification and transport, as well as the replication of other viruses. The last group is made up of the carboxylic ionophores, such as monensin, which exchange endocytic protons for cytoplasmic potassium and sodium. Among these categories, chloroquine and hydroxychloroquine have been investigated as possible treatments for COVID-19. However, despite demonstrating efficacy in preventing SARS-CoV-2 replication both in vitro and in animal models of COVID-19, these agents failed to produce significant clinical benefit in humans in large randomized controlled trials (RCTs) [157,158,159,160,161,162,163,164].

**Figure 3:**
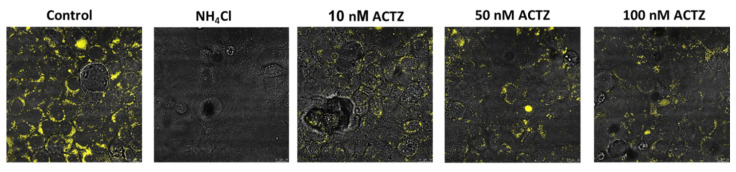
Acetazolamide interferes with lysosomal acidification. Acetazolamide (ACTZ) is a pan-carbonic anhydrase (CA) inhibitor. These enzymes are important in the catalysis of the dissociation of H_2_CO_3_ to HCO_3_^−^ and H^+^. Based on the activity of CA in endo/lysosomal compartments, we examined the effect of ACTZ on lysosomal pH using LysoSensor blue reagent. Treatment of Vero 6 cells with ACTZ at 50 nm significantly prevented the acidification of lysosomes. Both lower (10 nm) and well higher concentrations of ACTZ (100 nm) were able to significantly inhibit the acidification of lysosomes (Figure 3). (*Unpublished data from the author’s laboratory*).

A potential novel approach to inhibit lysosomal acidification could be via the use of acetazolamide (ACTZ), a potent carbonic anhydrase (CA) inhibitor. There are distinct CA isoforms, such as CA IX, that are expressed in the lysosomal compartment and may play a critical role in intra-lysosomal pH regulation. No prior experiments have specifically examined the role of CA inhibitors in lysosomal acidification. For this reason, we studied the role of acetazolamide on lysosomal acidification using Vero E6 cells. Our results demonstrated a significant inhibition of lysosomal acidification using physiologic concentrations of acetazolamide (Figure 3). Based on these results, we propose that ACTZ may be a potential therapeutic agent for inhibiting the uncoating and release of the viral genome from the late-stage endosome and serve as a possible treatment for early COVID-19 without the cardiotoxicity associated with hydroxychloroquine.

Whether or not treatments with inhibitors of endosomal acidification will prove clinically effective will depend on developing a better understanding of the course of SARS-CoV-2 infection and the stage of the disease at which interventions are the most effective. We posit that, similarly to other antiviral agents, inhibitors of endosomal acidification, such as acetazolamide, may be most effective in the treatment of respiratory disease in the very early symptomatic or pre-symptomatic stages of SARS-CoV-2 infection, when viral entry and endosomal processing of the virus can be meaningfully inhibited. Similarly, we speculate that prophylactic treatment with acetazolamide may prove effective in the prevention of direct, virally induced AKI, with timing of therapy and patient selection likely crucial to providing benefit. Acetazolamide must be used with caution in patients with respiratory acidosis or volume depletion. However, the vast majority of patients with respiratory failure from COVID-19 (in the absence of chronic pulmonary disease) present initially with hypoxemia and develop hypercarbia later in the course of disease, typically only after initiation of mechanical ventilation [165,166]. As such, the use of acetazolamide may be well tolerated from an acid–base perspective early in the course of COVID-19, when inhibition of viral cellular entry is more likely to be effective.

### 5.4. Potential Therapeutic Targets: Modulation of the Renin–Angiotensin–Aldosterone System

Given that ACE2 serves as a receptor for cellular viral entry, tremendous interest has been generated in the interaction between the renin–angiotensin–aldosterone system (RAAS) and the virus [167]. Initial concerns that the use of RAAS inhibitors, namely angiotensin-converting enzyme inhibitors (ACEi) or angiotensin-receptor blockers (ARB), may increase the risk or severity of COVID-19 via upregulation of ACE2 have been refuted [168,169,170], and the risk of harm from ACEi or ARB use in the setting of COVID-19 has been clearly disproven [171,172,173,174,175,176,177,178,179]. This has led to multiple RCTs of ACEi or ARB initiation for patients newly diagnosed with COVID-19, which are still largely underway or pending publication (NCT04311177, NCT04312009, NCT04345406, NCT04355429, NCT04366050) [180].

Further attempts to modulate the RAAS system have expanded beyond the traditional RAAS axis towards studies of the relatively novel ACE2/angiotensin-(1-7) axis. In contrast to angiotensin II, which has vasoconstrictive, proinflammatory, prothrombotic, and profibrotic effects, angiotensin-(1-7) has vasodilatory, anti-inflammatory, antithrombotic, and antifibrotic properties in multiple organs, including the lungs and kidneys [181,182]. Animal studies carried out before the pandemic suggested that the balance between angiotensin II and angiotensin-(1-7) may determine the outcome of acute lung injury from other viruses or non-viral causes, leading to a human pilot RCT in 2017 of recombinant human ACE2 (rhACE2) in ARDS [183,184,185,186,187].

The relationship between RAAS and lung injury, however, is clearly complex, as evidenced by a study of over 100 patients admitted with COVID-19, which demonstrated that lower angiotensin II levels were correlated with worsening lung damage and need for ICU admission [188]. Likewise, these results were subsequently replicated in another study using a distinct methodology, which found that the ratio of the plasma levels of angiotensin-(1-7) to angiotensin II was higher in 88 patients admitted with COVID-19 compared to 38 age- and sex-matched healthy controls [189]. This study and others also implicate a role for ADAM17 (a disintegrin and metalloproteinase 17), a membrane-bound protease that is upregulated in SARS-CoV and SARS-CoV-2 infections and cleaves membrane-bound ACE2, releasing it into the circulation and thereby potentially diminishing ACE2-mediated protection against angiotensin II tissue activity [189,190]. ADAM17 inhibition may therefore be protective in COVID-19, though additional studies are needed [191]. On the other hand, these two studies, which seemingly contradict the RCTs that have demonstrated RAAS inhibitor continuation is safe in COVID-19, demonstrate that the interplay between COVID-19 and the two counterbalancing axes of the RAAS system is clearly more complex than we currently understand.

In addition to lung injury, the RAAS may play an important role in the development of AKI in COVID-19. For example, in one study of 51 patients with COVID-19 admitted to a French ICU, over 50% (*n* = 26) developed AKI, and in comparison with non-AKI patients, the AKI patients exhibited features of activation of the traditional RAAS axis, with statistically higher serum renin and aldosterone concentrations [192].

In the case of COVID-19, exogenous soluble rhACE2 may decrease severity of illness both through the tissue-protective effects of angiotensin-(1-7) and by acting as a dummy receptor or virus-inactivating molecule and directly interfering with viral cellular uptake and viral replication, as was demonstrated in the in vitro study by Monteil et al. referenced above [145]. Multiple RCTs of soluble rhACE2 or other novel therapeutics aimed at restoring the balance between angiotensin II and angiotensin-(1-7) are currently underway (NCT04335136, NCT04401423, NCT04419610, NCT04924660, NCT02735707).

### 5.5. Potential Therapeutic Targets: Complement

Complement activation plays a central role in a variety of glomerular diseases, such as atypical hemolytic uremic syndrome (aHUS) and dense deposit disease, for which complement inhibitors, such as eculizumab, are being increasingly employed. As previously discussed, a substantial minority of patients with COVID-19-associated AKI appear, based on histopathology, to have glomerular microangiopathy reminiscent of aHUS. A possible role of complement activation in the pathogenesis of COVID-19-associated AKI is suggested by a French study in which elements of the complement system, particularly components of the alternative and lectin pathways, were found in kidney histopathologic specimens: six biopsies and three autopsies, from nine patients with COVID-19 and AKI, as well as histologic ATI [3]. The investigators also analyzed the proteinaceous material clogging plasma adsorbers of extracorporeal circuits used to treat patients with severe COVID-19 and found significant deposition of C3 and other peptides involved in complement activation [3]. Another study of autopsy specimens from 12 COVID-19 patients who died of respiratory failure found, despite minimal histopathological ATI, deposition of multiple components of the complement cascade in kidney tissue, especially in the tubules, vessels, and periglomerular regions, with only mild staining restricted to C5b-9 in the glomeruli [95]. Whether complement inhibition or modulation has a role in the treatment of COVID-19-associated AKI remains to be seen.

## 6. Discussion and Conclusions

### 6.1. Discussion

Approximately two years into the COVID-19 pandemic, while we still have many unanswered questions, we have an increasing understanding of the interplay between viral infection in COVID-19 and AKI. An overwhelming amount of data demonstrates that AKI afflicts a substantial number of patients with COVID-19 and is independently associated with poor overall outcomes. Likewise, there is no doubt that pre-existing kidney disease, including both ESKD and pre-dialysis CKD, greatly increases the risk of harm from COVID-19. However, there are also increasing data that suggest COVID-19 has lasting effects on the kidney, including an increased risk of long-term renal dysfunction—effects that are not fully explained by AKI diagnosed during the index infection. Though additional studies are needed, the accumulating data suggest that COVID-19-associated AKI is often caused, at least in part, by the direct effect of SARS-CoV-2 upon renal tissue.

Indeed, the molecular mechanisms of SARS-CoV-2 infection reviewed in this article are all associated with potential novel therapeutic targets for the treatment of COVID-19-associated AKI or COVID-19 in general. These targets include the steps of the viral cellular-entry process; components, both traditional and non-traditional, of the RAAS system; and elements of the complement system. For example, inhibitors of lysosomal acidification, a process essential to cellular infection, may hold promise for treatment of COVID-19. Specifically, while the antimalarial agent hydroxychloroquine is clearly ineffective, other potentially less toxic blockers of lysosomal acidification, such as acetazolamide, may be effective and warrant further study.

However, blockers of viral cellular entry similar to remdesivir and other antivirals are most likely to be effective early in the course of SARS-CoV-2 infection [153,154,193]. While the importance of introducing antiviral therapy early is also characteristic of other respiratory viral infections, such as influenza, it seems especially true of COVID-19, in which most patients who develop severe illness appear to progress from an early viral phase into a late hyperinflammatory or “cytokine storm” phase in which multiorgan dysfunction, including AKI, often develops [28,64,85,194,195]. The role of cytokine storm in severe COVID-19 and whether the syndrome is more relevant to COVID-19 than other types of critical illness remains a debated topic [196,197,198,199]. The most compelling evidence that a maladaptive immune response plays a fundamental role in the development of severe COVID-19 may be the fact that the only therapies shown to provide convincing benefit for the treatment of patients are immunomodulatory agents, including corticosteroids, IL-6 inhibitors, and Janus kinase inhibitors [200,201,202,203,204]. Notably, these trials of immunomodulatory therapy, specifically of dexamethasone and tocilizumab, demonstrated statistically significant renal benefit in the form of decreased need for RRT as secondary endpoints; however, whether this benefit is mediated by specific effects of these agents upon the kidneys or due to overall decreased severity of illness is unclear [200,203].

In this regard, modulators of the RAAS system may hold particular promise as potentially effective therapies throughout the course of COVID-19. First, they may potentially interfere with cellular viral entry by blocking the binding of the viral spike protein S1 domain and the membrane-bound ACE2 receptor. Second, RAAS modulators may prevent the progression from early infection to the life-threatening hyperinflammatory stage of COVID-19 by rebalancing the RAAS system towards higher angiotensin-(1-7) activity and lower angiotensin II activity, the latter of which may mediate harmful proinflammatory, prothrombotic, and profibrotic effects upon the kidneys, lungs, and other organs.

### 6.2. Conclusions

**Figure 4:**
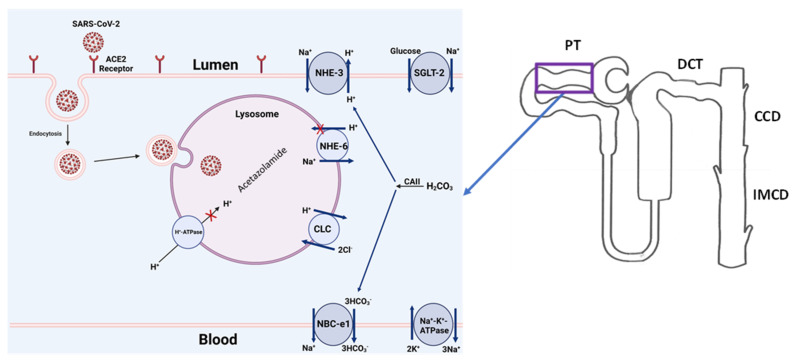
Binding of SARS-CoV-2 with the ACE2 receptor and entry into kidney proximal tubule cells. Proposed schematic diagram depicting the binding of SARS-CoV-2 spike protein to the apical membrane of the kidney proximal tubule cells, followed by the internalization of the virus through endocytosis and its entry into lysosomes. The crucial transporters responsible for lysosomal acidification are shown. The role of carbonic anhydrase inhibitors, such as acetazolamide, in impairing lysosomal acidification via interference with the activity of acid-importing H^+^-ATPase and NHE-6 is highlighted. The proximal-tubule-specific plasma membrane transporters are shown. *Image created with BioRender*. *PT*: *proximal tubule*; *DCT*: *distal convoluted tubule*; *CCD*: *cortical colleting duct*; *IMCD*: *inner medullary collecting duct*.

The debate about whether COVID-19-associated AKI is caused purely by indirect insults or is mediated, at least in part, by the direct effect of SARS-CoV-2 upon renal tissue is likely to continue [28,102]. However, the experimental studies outlined above, combined with the epidemiologic data presented, suggest the following: (1) kidney cells can be targeted by SARS-CoV-2 via the interaction of their surface receptor complexes, ACE2 and TMPRSS2; (2) SARS-CoV-2 can lead to renal epithelial injury via indirect and direct means; (3) direct viral damage to the renal epithelium may be the primary cause of AKI in some, but not all, cases of COVID-19-associated AKI; and (4) for many patients, including those with either subclinical or clinically overt AKI, SARS-CoV-2 may aggravate or perpetuate kidney injury initiated by non-viral causes. The schematic diagram in Figure 4 summarizes the above steps by demonstrating the binding of SARS-CoV-2 with the ACE2 receptor on the apical membrane of kidney proximal tubule cells, followed by the entry of the virus into the cell via endocytosis. Once inside the cell, the COVID-19 virus crosses into the lysosome, where the acidic pH-dependent endosomal proteases cleave the viral glycoprotein segments to prepare the virus for replication and propagation. Disrupting any of the above processes (binding with ACE-2, inhibiting the endocytosis, and interrupting the endosomal acidification) can potentially prevent kidney-tubule damage from COVID-19.

As we have all witnessed over the last 12 months, the introduction of highly effective vaccines for COVID-19, while vital to mitigating further widespread loss of life, has not brought about the rapid end of the COVID-19 pandemic as many had hoped. Though the virus and its spread remain as unpredictable as ever, with new SARS-CoV-2 variants emerging monthly, experts are increasingly predicting a transition from a pandemic to endemic COVID-19 [205]. As such, the ongoing study of the molecular mechanisms of SARS-CoV-2 infection, including those described in this review, remains as important as ever as we continue to strive to develop effective treatments for COVID-19 and its major complications, including AKI.

## 7. Material and Methods

### 7.1. Materials

Spike protein fragment S1 tagged with IgG1 Fc (S1. Fc) was purchased from SinoBiological (Wayne, PA, USA). Protein-G-conjugated green fluorescent protein beads were purchased from Spherotech (Lake Forest, IL, USA). LySosensor blue DND-167 was purchased from Thermofisher Scientific (Waltham, MA, USA). Acetazolamide was purchased from Sigma-Aldrich (St. Louis, MO, USA).

### 7.2. Immunofluorescence Microscopy

Human kidney sections were fixed in 4% paraformaldehyde in phosphate-buffered saline (PBS) for 24 h at 4 degrees C, then transferred to 70% ethanol for the same period of time and at the same temperature. Fixed samples were paraffin-embedded, cut into 5 mm sections, and stored until used. To prepare for immunofluorescence staining, slides were baked at 60 °C for 1.5 h and underwent citrate antigen retrieval. Next, slides were incubated with the primary antibodies overnight in a humidity chamber at 4 °C. The following day, slides were incubated in secondary antibodies (AlexaFluor 594 or 488, Invitrogen, Waltham, MA, USA) in a humidity chamber at room temperature for 2 h. Slides were washed in PBS and, after drying, were cover-slipped with Vectashield Hard Set (Vector Labs, Burlingame, CA, USA). The expression of ACE2 (antibody purchased from R & D Systems) and NBCe-1 (antibody generated by our lab) was examined using a Zeiss Airyscan (Zeiss, Dublin, CA, USA). M2 microscopy and images were obtained via Zen Software 2.3 (Zeiss, Dublin, CA, USA).

### 7.3. S1 Fragment Binding to Vero 6 Cells

Binding of the S1 fragment of spike protein to Vero 6 cells was examined using S1-tagged IgG1 Fc recombinant protein adsorbed to green fluorescent protein (GFP) beads-coated with protein G (S1. Fc-GFP). Fluorescent beads conjugated with protein G were washed in PBS with 1% BSA and incubated overnight at 4 °C with S1.Fc. The following day, beads were washed in PBS with 1% BSA, blocked with PBS with 4% BSA overnight at 4 °C, and washed again with PBS with 1% BSA. For the binding assay, 90% confluent monolayers of Vero E6 cells were washed twice using PBS with 1% BSA, incubated with S1. Fc adsorbed to GFP-protein-G-coated beads (S1. Fc-GFP) in PBS with 1% BSA overnight at 4 °C, and washed with PBS with 1% BSA before fixation with 4% paraformaldehyde in PBS. Staining was performed in the presence or absence of DAPI (4′,6-diamidino-2-phenylindole), a fluorescent marker of nuclei. Binding of S1. Fc-GFP and Fc-GFP (with no S1 component) to Vero E6 cells was visualized by a Zeiss Airyscan microscope utilizing Zen 2.3 software.

### 7.4. Effect of Acetazolamide on Lysosomal Acidification

In vitro studies suggest that treatments that interfere with lysosome acidification suppress the infectivity of SARS-CoV-2 in Vero 6 cells (text). The effect of inhibition of CA with ACTZ on lysosomal pH was examined using LysoSensor blue reagent. Vero E6 monolayers were grown to confluence, incubated in complete medium, and treated with 20 µm ammonium chloride (NH_4_Cl) or ACTZ (10, 50, and 100 nm) for 24 h. Lysosomal acidification (presence of yellow fluorescence) was assessed using LysoSensor blue DND-167. Untreated cells showed significant fluorescent signaling, indicating the presence of acidified lysosomes (HBSS), whereas treatment with NH_4_Cl completely abrogated lysosomal acidification. Treatment with increasing ACTZ levels significantly inhibited the acidification of lysosomes.

## Data Availability

All data generated in our laboratory is available to other investigators upon request.

## References

[1] Johns Hopkins University of Medicine Coronavirus Resource Center. https://coronavirus.jhu.edu/.

[2] Nadim M.K., Forni L.G., Mehta R.L., Connor M.J., Liu K.D., Ostermann M., Rimmele T., Zarbock A., Bell S., Bihorac A. (2020). COVID-19-associated acute kidney injury: Consensus report of the 25th Acute Disease Quality Initiative (ADQI) Workgroup. Nat. Rev. Nephrol..

[3] Pfister F., Vonbrunn E., Ries T., Jack H.M., Uberla K., Lochnit G., Sheriff A., Herrmann M., Buttner-Herold M., Amann K. (2020). Complement Activation in Kidneys of Patients with COVID-19. Front. Immunol..

[4] Hoste E.A., Bagshaw S.M., Bellomo R., Cely C.M., Colman R., Cruz D.N., Edipidis K., Forni L.G., Gomersall C.D., Govil D. (2015). Epidemiology of acute kidney injury in critically ill patients: The multinational AKI-EPI study. Intensive Care Med..

[5] Hoste E.A.J., Kellum J.A., Selby N.M., Zarbock A., Palevsky P.M., Bagshaw S.M., Goldstein S.L., Cerda J., Chawla L.S. (2018). Global epidemiology and outcomes of acute kidney injury. Nat. Rev. Nephrol..

[6] Chen N., Zhou M., Dong X., Qu J., Gong F., Han Y., Qiu Y., Wang J., Liu Y., Wei Y. (2020). Epidemiological and clinical characteristics of 99 cases of 2019 novel coronavirus pneumonia in Wuhan, China: A descriptive study. Lancet.

[7] Cheng Y., Luo R., Wang K., Zhang M., Wang Z., Dong L., Li J., Yao Y., Ge S., Xu G. (2020). Kidney disease is associated with in-hospital death of patients with COVID-19. Kidney Int..

[8] Guan W.J., Ni Z.Y., Hu Y., Liang W.H., Ou C.Q., He J.X., Liu L., Shan H., Lei C.L., Hui D.S.C. (2020). Clinical Characteristics of Coronavirus Disease 2019 in China. N. Engl. J. Med..

[9] Wang W., Xu Y., Gao R., Lu R., Han K., Wu G., Tan W. (2020). Detection of SARS-CoV-2 in Different Types of Clinical Specimens. JAMA.

[10] Wang L., Li X., Chen H., Yan S., Li D., Li Y., Gong Z. (2020). Coronavirus Disease 19 Infection Does Not Result in Acute Kidney Injury: An Analysis of 116 Hospitalized Patients from Wuhan, China. Am. J. Nephrol..

[11] Huang C., Wang Y., Li X., Ren L., Zhao J., Hu Y., Zhang L., Fan G., Xu J., Gu X. (2020). Clinical features of patients infected with 2019 novel coronavirus in Wuhan, China. Lancet.

[12] Yang X., Yu Y., Xu J., Shu H., Xia J., Liu H., Wu Y., Zhang L., Yu Z., Fang M. (2020). Clinical course and outcomes of critically ill patients with SARS-CoV-2 pneumonia in Wuhan, China: A single-centered, retrospective, observational study. Lancet Respir. Med..

[13] Chen T., Wu D., Chen H., Yan W., Yang D., Chen G., Ma K., Xu D., Yu H., Wang H. (2020). Clinical characteristics of 113 deceased patients with coronavirus disease 2019: Retrospective study. BMJ.

[14] Lin L., Wang X., Ren J., Sun Y., Yu R., Li K., Zheng L., Yang J. (2020). Risk factors and prognosis for COVID-19-induced acute kidney injury: A meta-analysis. BMJ Open.

[15] Hirsch J.S., Ng J.H., Ross D.W., Sharma P., Shah H.H., Barnett R.L., Hazzan A.D., Fishbane S., Jhaveri K.D., Northwell COVID-19 Research Consortium (2020). Acute kidney injury in patients hospitalized with COVID-19. Kidney Int..

[16] Chan L., Chaudhary K., Saha A., Chauhan K., Vaid A., Zhao S., Paranjpe I., Somani S., Richter F., Miotto R. (2021). AKI in Hospitalized Patients with COVID-19. J. Am. Soc. Nephrol..

[17] Mohamed M.M.B., Lukitsch I., Torres-Ortiz A.E., Walker J.B., Varghese V., Hernandez-Arroyo C.F., Alqudsi M., LeDoux J.R., Velez J.C.Q. (2020). Acute Kidney Injury Associated with Coronavirus Disease 2019 in Urban New Orleans. Kidney360.

[18] Gupta S., Coca S.G., Chan L., Melamed M.L., Brenner S.K., Hayek S.S., Sutherland A., Puri S., Srivastava A., Leonberg-Yoo A. (2021). AKI Treated with Renal Replacement Therapy in Critically Ill Patients with COVID-19. J. Am. Soc. Nephrol..

[19] Fisher M., Neugarten J., Bellin E., Yunes M., Stahl L., Johns T.S., Abramowitz M.K., Levy R., Kumar N., Mokrzycki M.H. (2020). AKI in Hospitalized Patients with and without COVID-19: A Comparison Study. J. Am. Soc. Nephrol..

[20] Bowe B., Cai M., Xie Y., Gibson A.K., Maddukuri G., Al-Aly Z. (2020). Acute Kidney Injury in a National Cohort of Hospitalized US Veterans with COVID-19. Clin. J. Am. Soc. Nephrol..

[21] Kolhe N.V., Fluck R.J., Selby N.M., Taal M.W. (2020). Acute kidney injury associated with COVID-19: A retrospective cohort study. PLoS Med..

[22] Rubin S., Orieux A., Prevel R., Garric A., Bats M.L., Dabernat S., Camou F., Guisset O., Issa N., Mourissoux G. (2020). Characterization of acute kidney injury in critically ill patients with severe coronavirus disease 2019. Clin. Kidney J..

[23] Fominskiy E.V., Scandroglio A.M., Monti G., Calabro M.G., Landoni G., Dell’Acqua A., Beretta L., Moizo E., Ravizza A., Monaco F. (2021). Prevalence, Characteristics, Risk Factors, and Outcomes of Invasively Ventilated COVID-19 Patients with Acute Kidney Injury and Renal Replacement Therapy. Blood Purif..

[24] Husain-Syed F., Wilhelm J., Kassoumeh S., Birk H.W., Herold S., Vadasz I., Walmrath H.D., Kellum J.A., Ronco C., Seeger W. (2020). Acute kidney injury and urinary biomarkers in hospitalized patients with coronavirus disease-2019. Nephrol. Dial. Transplant..

[25] Chaibi K., Dao M., Pham T., Gumucio-Sanguino V.D., Di Paolo F.A., Pavot A., Cohen Y., Dreyfuss D., Perez-Fernandez X., Gaudry S. (2020). Severe Acute Kidney Injury in Patients with COVID-19 and Acute Respiratory Distress Syndrome. Am. J. Respir. Crit. Care Med..

[26] Charytan D.M., Parnia S., Khatri M., Petrilli C.M., Jones S., Benstein J., Horwitz L.I. (2021). Decreasing Incidence of Acute Kidney Injury in Patients with COVID-19 Critical Illness in New York City. Kidney Int. Rep..

[27] Husain-Syed F., Birk H.W., Ronco C. (2021). Coronavirus Disease 2019 and Acute Kidney Injury: What Have We Learned?. Kidney Int. Rep..

[28] Legrand M., Bell S., Forni L., Joannidis M., Koyner J.L., Liu K., Cantaluppi V. (2021). Pathophysiology of COVID-19-associated acute kidney injury. Nat. Rev. Nephrol..

[29] Silver S.A., Beaubien-Souligny W., Shah P.S., Harel S., Blum D., Kishibe T., Meraz-Munoz A., Wald R., Harel Z. (2021). The Prevalence of Acute Kidney Injury in Patients Hospitalized with COVID-19 Infection: A Systematic Review and Meta-analysis. Kidney Med..

[30] Pelayo J., Lo K.B., Bhargav R., Gul F., Peterson E., DeJoy Iii R., Salacup G.F., Albano J., Gopalakrishnan A., Azmaiparashvili Z. (2020). Clinical Characteristics and Outcomes of Community- and Hospital-Acquired Acute Kidney Injury with COVID-19 in a US Inner City Hospital System. Cardiorenal Med..

[31] Pei G., Zhang Z., Peng J., Liu L., Zhang C., Yu C., Ma Z., Huang Y., Liu W., Yao Y. (2020). Renal Involvement and Early Prognosis in Patients with COVID-19 Pneumonia. J. Am. Soc. Nephrol..

[32] Chaudhri I., Moffitt R., Taub E., Annadi R.R., Hoai M., Bolotova O., Yoo J., Dhaliwal S., Sahib H., Daccueil F. (2020). Association of Proteinuria and Hematuria with Acute Kidney Injury and Mortality in Hospitalized Patients with COVID-19. Kidney Blood Press. Res..

[33] Paek J.H., Kim Y., Park W.Y., Jin K., Hyun M., Lee J.Y., Kim H.A., Kwon Y.S., Park J.S., Han S. (2020). Severe acute kidney injury in COVID-19 patients is associated with in-hospital mortality. PLoS ONE.

[34] Cheng Y., Luo R., Wang X., Wang K., Zhang N., Zhang M., Wang Z., Dong L., Li J., Zeng R. (2020). The Incidence, Risk Factors, and Prognosis of Acute Kidney Injury in Adult Patients with Coronavirus Disease 2019. Clin. J. Am. Soc. Nephrol..

[35] Ng J.H., Hirsch J.S., Hazzan A., Wanchoo R., Shah H.H., Malieckal D.A., Ross D.W., Sharma P., Sakhiya V., Fishbane S. (2021). Outcomes among Patients Hospitalized with COVID-19 and Acute Kidney Injury. Am. J. Kidney Dis..

[36] Perez Ingles D., Illescas A., Perryman Collins N., Jordyn A.N., Marinaro J.L., Argyropoulos C., Teixeira J.P. (2021). Impact of COVID-19 Pandemic on Crude Mortality Rates Associated with Acute Kidney Injury Requiring Continuous Renal Replacement Therapy: A Single-Center Study [abstract]. Am. J. Respir. Crit. Care Med..

[37] Russo E., Esposito P., Taramasso L., Magnasco L., Saio M., Briano F., Russo C., Dettori S., Vena A., Di Biagio A. (2021). Kidney disease and all-cause mortality in patients with COVID-19 hospitalized in Genoa, Northern Italy. J. Nephrol..

[38] Domecq J.P., Lal A., Sheldrick C.R., Kumar V.K., Boman K., Bolesta S., Bansal V., Harhay M.O., Garcia M.A., Kaufman M. (2021). Outcomes of Patients with Coronavirus Disease 2019 Receiving Organ Support Therapies: The International Viral Infection and Respiratory Illness Universal Study Registry. Crit. Care Med..

[39] Liu Y.F., Zhang Z., Pan X.L., Xing G.L., Zhang Y., Liu Z.S., Tu S.H. (2021). The chronic kidney disease and acute kidney injury involvement in COVID-19 pandemic: A systematic review and meta-analysis. PLoS ONE.

[40] Coca A., Burballa C., Centellas-Perez F.J., Perez-Saez M.J., Bustamante-Munguira E., Ortega A., Duenas C., Arenas M.D., Perez-Martinez J., Ruiz G. (2020). Outcomes of COVID-19 among Hospitalized Patients with Non-dialysis CKD. Front. Med..

[41] Henry B.M., Lippi G. (2020). Chronic kidney disease is associated with severe coronavirus disease 2019 (COVID-19) infection. Int. Urol. Nephrol..

[42] Williamson E.J., Walker A.J., Bhaskaran K., Bacon S., Bates C., Morton C.E., Curtis H.J., Mehrkar A., Evans D., Inglesby P. (2020). Factors associated with COVID-19-related death using OpenSAFELY. Nature.

[43] Ioannou G.N., Locke E., Green P., Berry K., O’Hare A.M., Shah J.A., Crothers K., Eastment M.C., Dominitz J.A., Fan V.S. (2020). Risk Factors for Hospitalization, Mechanical Ventilation, or Death among 10131 US Veterans with SARS-CoV-2 Infection. JAMA Netw. Open.

[44] Portoles J., Marques M., Lopez-Sanchez P., de Valdenebro M., Munez E., Serrano M.L., Malo R., Garcia E., Cuervas V. (2020). Chronic kidney disease and acute kidney injury in the COVID-19 Spanish outbreak. Nephrol. Dial. Transplant..

[45] Brogan M., Ross M.J. (2022). The Impact of Chronic Kidney Disease on Outcomes of Patients with COVID-19 Admitted to the Intensive Care Unit. Nephron.

[46] Pakhchanian H., Raiker R., Mukherjee A., Khan A., Singh S., Chatterjee A. (2021). Outcomes of COVID-19 in CKD Patients: A Multicenter Electronic Medical Record Cohort Study. Clin. J. Am. Soc. Nephrol..

[47] Gibertoni D., Reno C., Rucci P., Fantini M.P., Buscaroli A., Mosconi G., Rigotti A., Giudicissi A., Mambelli E., Righini M. (2021). COVID-19 incidence and mortality in non-dialysis chronic kidney disease patients. PLoS ONE.

[48] Flythe J.E., Assimon M.M., Tugman M.J., Chang E.H., Gupta S., Shah J., Sosa M.A., Renaghan A.D., Melamed M.L., Wilson F.P. (2021). Characteristics and Outcomes of Individuals with Pre-existing Kidney Disease and COVID-19 Admitted to Intensive Care Units in the United States. Am. J. Kidney Dis..

[49] Ozturk S., Turgutalp K., Arici M., Gok M., Islam M., Altiparmak M.R., Aydin Z., Doner B., Eren N., Sengul E. (2021). Characteristics and outcomes of hospitalised older patients with chronic kidney disease and COVID-19: A multicenter nationwide controlled study. Int. J. Clin. Pract..

[50] Ozturk S., Turgutalp K., Arici M., Odabas A.R., Altiparmak M.R., Aydin Z., Cebeci E., Basturk T., Soypacaci Z., Sahin G. (2020). Mortality analysis of COVID-19 infection in chronic kidney disease, haemodialysis and renal transplant patients compared with patients without kidney disease: A nationwide analysis from Turkey. Nephrol. Dial. Transplant..

[51] Yang D., Xiao Y., Chen J., Chen Y., Luo P., Liu Q., Yang C., Xiong M., Zhang Y., Liu X. (2020). COVID-19 and chronic renal disease: Clinical characteristics and prognosis. QJM.

[52] Iaccarino G., Grassi G., Borghi C., Ferri C., Salvetti M., Volpe M. (2020). SARS-RAS Investigators, Age and Multimorbidity Predict Death among COVID-19 Patients: Results of the SARS-RAS Study of the Italian Society of Hypertension. Hypertension.

[53] Oetjens M.T., Luo J.Z., Chang A., Leader J.B., Hartzel D.N., Moore B.S., Strande N.T., Kirchner H.L., Ledbetter D.H., Justice A.E. (2020). Electronic health record analysis identifies kidney disease as the leading risk factor for hospitalization in confirmed COVID-19 patients. PLoS ONE.

[54] Carlson N., Nelveg-Kristensen K.E., Freese Ballegaard E., Feldt-Rasmussen B., Hornum M., Kamper A.L., Gislason G., Torp-Pedersen C. (2021). Increased vulnerability to COVID-19 in chronic kidney disease. J. Intern. Med..

[55] Bowe B., Xie Y., Xu E., Al-Aly Z. (2021). Kidney Outcomes in Long COVID. J. Am. Soc. Nephrol..

[56] Al-Aly Z., Xie Y., Bowe B. (2021). High-dimensional characterization of post-acute sequelae of COVID-19. Nature.

[57] Huang C., Huang L., Wang Y., Li X., Ren L., Gu X., Kang L., Guo L., Liu M., Zhou X. (2021). 6-month consequences of COVID-19 in patients discharged from hospital: A cohort study. Lancet.

[58] Daugherty S.E., Guo Y., Heath K., Dasmarinas M.C., Jubilo K.G., Samranvedhya J., Lipsitch M., Cohen K. (2021). Risk of clinical sequelae after the acute phase of SARS-CoV-2 infection: Retrospective cohort study. BMJ.

[59] Nugent J., Aklilu A., Yamamoto Y., Simonov M., Li F., Biswas A., Ghazi L., Greenberg H., Mansour G., Moledina G. (2021). Assessment of Acute Kidney Injury and Longitudinal Kidney Function after Hospital Discharge among Patients with and without COVID-19. JAMA Netw. Open.

[60] Su H., Yang M., Wan C., Yi L.X., Tang F., Zhu H.Y., Yi F., Yang H.C., Fogo A.B., Nie X. (2020). Renal histopathological analysis of 26 postmortem findings of patients with COVID-19 in China. Kidney Int..

[61] Golmai P., Larsen C.P., DeVita M.V., Wahl S.J., Weins A., Rennke H.G., Bijol V., Rosenstock J.L. (2020). Histopathologic and Ultrastructural Findings in Postmortem Kidney Biopsy Material in 12 Patients with AKI and COVID-19. J. Am. Soc. Nephrol..

[62] Alexander M.P., Mangalaparthi K.K., Madugundu A.K., Moyer A.M., Adam B.A., Mengel M., Singh S., Herrmann S.M., Rule A.D., Cheek E.H. (2021). Acute Kidney Injury in Severe COVID-19 Has Similarities to Sepsis-Associated Kidney Injury: A Multi-Omics Study. Mayo Clin. Proc..

[63] Vasquez-Bonilla W.O., Orozco R., Argueta V., Sierra M., Zambrano L.I., Munoz-Lara F., Lopez-Molina D.S., Arteaga-Livias K., Grimes Z., Bryce C. (2020). A review of the main histopathological findings in coronavirus disease 2019. Hum. Pathol..

[64] Xia P., Wen Y., Duan Y., Su H., Cao W., Xiao M., Ma J., Zhou Y., Chen G., Jiang W. (2020). Clinicopathological Features and Outcomes of Acute Kidney Injury in Critically Ill COVID-19 with Prolonged Disease Course: A Retrospective Cohort. J. Am. Soc. Nephrol..

[65] Nasr S.H., Alexander M.P., Cornell L.D., Herrera L.H., Fidler M.E., Said S.M., Zhang P., Larsen C.P., Sethi S. (2021). Kidney Biopsy Findings in Patients with COVID-19, Kidney Injury, and Proteinuria. Am. J. Kidney Dis..

[66] Wu H., Larsen C.P., Hernandez-Arroyo C.F., Mohamed M.M.B., Caza T., Sharshir M., Chughtai A., Xie L., Gimenez J.M., Sandow T.A. (2020). AKI and Collapsing Glomerulopathy Associated with COVID-19 and APOL 1 High-Risk Genotype. J. Am. Soc. Nephrol..

[67] Kudose S., Batal I., Santoriello D., Xu K., Barasch J., Peleg Y., Canetta P., Ratner L.E., Marasa M., Gharavi A.G. (2020). Kidney Biopsy Findings in Patients with COVID-19. J. Am. Soc. Nephrol..

[68] Larsen C.P., Bourne T.D., Wilson J.D., Saqqa O., Sharshir M.A. (2020). Collapsing Glomerulopathy in a Patient with COVID-19. Kidney Int. Rep..

[69] Ferlicot S., Jamme M., Gaillard F., Oniszczuk J., Couturier A., May O., Grunenwald A., Sannier A., Moktefi A., Le Monnier O. (2021). The spectrum of kidney biopsies in hospitalized patients with COVID-19, acute kidney injury, and/or proteinuria. Nephrol. Dial. Transplant..

[70] Sharma P., Uppal N.N., Wanchoo R., Shah H.H., Yang Y., Parikh R., Khanin Y., Madireddy V., Larsen C.P., Jhaveri K.D. (2020). COVID-19-Associated Kidney Injury: A Case Series of Kidney Biopsy Findings. J. Am. Soc. Nephrol..

[71] May R.M., Cassol C., Hannoudi A., Larsen C.P., Lerma E.V., Haun R.S., Braga J.R., Hassen S.I., Wilson J., VanBeek C. (2021). A multi-center retrospective cohort study defines the spectrum of kidney pathology in Coronavirus 2019 Disease (COVID-19). Kidney Int..

[72] Akilesh S., Nast C.C., Yamashita M., Henriksen K., Charu V., Troxell M.L., Kambham N., Bracamonte E., Houghton D., Ahmed N.I. (2021). Multicenter Clinicopathologic Correlation of Kidney Biopsies Performed in COVID-19 Patients Presenting with Acute Kidney Injury or Proteinuria. Am. J. Kidney Dis..

[73] Hanley B., Naresh K.N., Roufosse C., Nicholson A.G., Weir J., Cooke G.S., Thursz M., Manousou P., Corbett R., Goldin R. (2020). Histopathological findings and viral tropism in UK patients with severe fatal COVID-19: A post-mortem study. Lancet Microbe.

[74] Schurink B., Roos E., Radonic T., Barbe E., Bouman C.S.C., de Boer H.H., de Bree G.J., Bulle E.B., Aronica E.M., Florquin S. (2020). Viral presence and immunopathology in patients with lethal COVID-19: A prospective autopsy cohort study. Lancet Microbe.

[75] Jhaveri K.D., Meir L.R., Flores Chang B.S., Parikh R., Wanchoo R., Barilla-LaBarca M.L., Bijol V., Hajizadeh N. (2020). Thrombotic microangiopathy in a patient with COVID-19. Kidney Int..

[76] El Shamy O., Munoz-Casablanca N., Coca S., Sharma S., Lookstein R., Uribarri J. (2021). Bilateral Renal Artery Thrombosis in a Patient with COVID-19. Kidney Med..

[77] Hernandez-Arroyo C.F., Varghese V., Mohamed M.M.B., Velez J.C.Q. (2020). Urinary Sediment Microscopy in Acute Kidney Injury Associated with COVID-19. Kidney360.

[78] Johnson A.C.M., Zager R.A. (2018). Mechanisms Underlying Increased TIMP2 and IGFBP7 Urinary Excretion in Experimental AKI. J. Am. Soc. Nephrol..

[79] Werion A., Belkhir L., Perrot M., Schmit G., Aydin S., Chen Z., Penaloza A., De Greef J., Yildiz H., Pothen L. (2020). SARS-CoV-2 causes a specific dysfunction of the kidney proximal tubule. Kidney Int..

[80] Kormann R., Jacquot A., Alla A., Corbel A., Koszutski M., Voirin P., Garcia Parrilla M., Bevilacqua S., Schvoerer E., Gueant J.L. (2020). Coronavirus disease 2019: Acute Fanconi syndrome precedes acute kidney injury. Clin. Kidney J..

[81] Morell-Garcia D., Ramos-Chavarino D., Bauca J.M., Argente Del Castillo P., Ballesteros-Vizoso M.A., Garcia de Guadiana-Romualdo L., Gomez-Cobo C., Pou J.A., Amezaga-Menendez R., Alonso-Fernandez A. (2021). Urine biomarkers for the prediction of mortality in COVID-19 hospitalized patients. Sci. Rep..

[82] Sundaram S., Soni M., Annigeri R. (2021). Urine abnormalities predict acute kidney injury in COVID-19 patients: An analysis of 110 cases in Chennai, South India. Diabetes Metab. Syndr..

[83] Caceres P.S., Savickas G., Murray S.L., Umanath K., Uduman J., Yee J., Liao T.D., Bolin S., Levin A.M., Khan M.N. (2021). High SARS-CoV-2 Viral Load in Urine Sediment Correlates with Acute Kidney Injury and Poor COVID-19 Outcome. J. Am. Soc. Nephrol..

[84] Omer D., Pleniceanu O., Gnatek Y., Namestnikov M., Cohen-Zontag O., Goldberg S., Friedman Y.E., Friedman N., Mandelboim M., Vitner E.B. (2021). Human Kidney Spheroids and Monolayers Provide Insights into SARS-CoV-2 Renal Interactions. J. Am. Soc. Nephrol..

[85] Peng S., Wang H.Y., Sun X., Li P., Ye Z., Li Q., Wang J., Shi X., Liu L., Yao Y. (2020). Early versus late acute kidney injury among patients with COVID-19-a multicenter study from Wuhan, China. Nephrol. Dial. Transplant..

[86] Khan S., Chen L., Yang C.R., Raghuram V., Khundmiri S.J., Knepper M.A. (2020). Does SARS-CoV-2 Infect the Kidney?. J. Am. Soc. Nephrol..

[87] Sang L., Chen S., Zheng X., Guan W., Zhang Z., Liang W., Zhong M., Jiang L., Pan C., Zhang W. (2020). The incidence, risk factors and prognosis of acute kidney injury in severe and critically ill patients with COVID-19 in mainland China: A retrospective study. BMC Pulm. Med..

[88] Van den Akker J.P., Egal M., Groeneveld A.B. (2013). Invasive mechanical ventilation as a risk factor for acute kidney injury in the critically ill: A systematic review and meta-analysis. Crit. Care.

[89] Kuiper J.W., Groeneveld A.B., Slutsky A.S., Plotz F.B. (2005). Mechanical ventilation and acute renal failure. Crit. Care Med..

[90] Strom T., Johansen R.R., Prahl J.O., Toft P. (2011). Sedation and renal impairment in critically ill patients: A post hoc analysis of a randomized trial. Crit. Care.

[91] Joannidis M., Druml W., Forni L.G., Groeneveld A.B.J., Honore P.M., Hoste E., Ostermann M., Oudemans-van Straaten H.M., Schetz M. (2017). Prevention of acute kidney injury and protection of renal function in the intensive care unit: Update 2017: Expert opinion of the Working Group on Prevention, AKI section, European Society of Intensive Care Medicine. Intensive Care Med..

[92] Fogagnolo A., Grasso S., Dres M., Gesualdo L., Murgolo F., Morelli E., Ottaviani I., Marangoni E., Volta C.A., Spadaro S. (2021). Focus on renal blood flow in mechanically ventilated patients with SARS-CoV-2: A prospective pilot study. J. Clin. Monit. Comput..

[93] Bhasin B., Veitla V., Dawson A.Z., Garacci Z., Sturgill D., Ozieh M.N., Regner K.R. (2021). AKI in Hospitalized Patients with COVID-19 and Seasonal Influenza: A Comparative Analysis. Kidney360.

[94] Taha M., Sano D., Hanoudi S., Esber Z., Elahi M., Gabali A., Chopra T., Draghici S., Samavati L. (2021). Platelets and renal failure in the SARS-CoV-2 syndrome. Platelets.

[95] Macor P., Durigutto P., Mangogna A., Bussani R., De Maso L., D’Errico S., Zanon M., Pozzi N., Meroni P.L., Tedesco F. (2021). Multiple-Organ Complement Deposition on Vascular Endothelium in COVID-19 Patients. Biomedicines.

[96] Vijayan A., Humphreys B.D. (2020). SARS-CoV-2 in the kidney: Bystander or culprit?. Nat. Rev. Nephrol..

[97] Batlle D., Soler M.J., Sparks M.A., Hiremath S., South A.M., Welling P.A., Swaminathan S. (2020). COVID-19 and ACE2 in Cardiovascular, Lung, and Kidney Working Group, Acute Kidney Injury in COVID-19: Emerging Evidence of a Distinct Pathophysiology. J. Am. Soc. Nephrol..

[98] Farkash E.A., Wilson A.M., Jentzen J.M. (2020). Ultrastructural Evidence for Direct Renal Infection with SARS-CoV-2. J. Am. Soc. Nephrol..

[99] Couturier A., Ferlicot S., Chevalier K., Guillet M., Essig M., Jaureguiberry S., Collarino R., Dargelos M., Michaut A., Geri G. (2020). Indirect effects of severe acute respiratory syndrome coronavirus 2 on the kidney in coronavirus disease patients. Clin. Kidney J..

[100] Rossi G.M., Delsante M., Pilato F.P., Gnetti L., Gabrielli L., Rossini G., Re M.C., Cenacchi G., Affanni P., Colucci M.E. (2020). Kidney Biopsy Findings in a Critically Ill COVID-19 Patient with Dialysis-Dependent Acute Kidney Injury: A Case Against “SARS-CoV-2 Nephropathy”. Kidney Int. Rep..

[101] Smith K.D., Akilesh S., Alpers C.E., Nicosia R.F. (2020). Am I a coronavirus?. Kidney Int..

[102] Roufosse C., Curtis E., Moran L., Hollinshead M., Cook T., Hanley B., Horsfield C., Neil D. (2020). Electron microscopic investigations in COVID-19: Not all crowns are coronas. Kidney Int..

[103] Calomeni E., Satoskar A., Ayoub I., Brodsky S., Rovin B.H., Nadasdy T. (2020). Multivesicular bodies mimicking SARS-CoV-2 in patients without COVID-19. Kidney Int..

[104] Miller S.E., Brealey J.K. (2020). Visualization of putative coronavirus in kidney. Kidney Int..

[105] Cassol C.A., Gokden N., Larsen C.P., Bourne T.D. (2020). Appearances Can Be Deceiving—Viral-like Inclusions in COVID-19 Negative Renal Biopsies by Electron Microscopy. Kidney360.

[106] Diao B., Wang C., Wang R., Feng Z., Zhang J., Yang H., Tan Y., Wang H., Wang C., Liu L. (2021). Human kidney is a target for novel severe acute respiratory syndrome coronavirus 2 infection. Nat. Commun..

[107] Frithiof R., Bergqvist A., Jarhult J.D., Lipcsey M., Hultstrom M. (2020). Presence of SARS-CoV-2 in urine is rare and not associated with acute kidney injury in critically ill COVID-19 patients. Crit. Care.

[108] Hassler L., Reyes F., Sparks M.A., Welling P., Batlle D. (2021). Evidence for and Against Direct Kidney Infection by SARS-CoV-2 in Patients with COVID-19. Clin. J. Am. Soc. Nephrol..

[109] Zhang Y.M., Zhang H. (2020). Genetic Roadmap for Kidney Involvement of Severe Acute Respiratory Syndrome Coronavirus 2 (SARS-CoV-2) Infection. Clin. J. Am. Soc. Nephrol..

[110] Pan X.W., Xu D., Zhang H., Zhou W., Wang L.H., Cui X.G. (2020). Identification of a potential mechanism of acute kidney injury during the COVID-19 outbreak: A study based on single-cell transcriptome analysis. Intensive Care Med..

[111] Hamming I., Timens W., Bulthuis M.L., Lely A.T., Navis G., van Goor H. (2004). Tissue distribution of ACE2 protein, the functional receptor for SARS coronavirus. A first step in understanding SARS pathogenesis. J. Pathol..

[112] Donoghue M., Hsieh F., Baronas E., Godbout K., Gosselin M., Stagliano N., Donovan M., Woolf B., Robison K., Jeyaseelan R. (2000). A novel angiotensin-converting enzyme-related carboxypeptidase (ACE2) converts angiotensin I to angiotensin 1-9. Circ. Res..

[113] Lin W., Fan J., Hu L.F., Zhang Y., Ooi J.D., Meng T., Jin P., Ding X., Peng L.K., Song L. (2021). Single-cell analysis of angiotensin-converting enzyme II expression in human kidneys and bladders reveals a potential route of 2019 novel coronavirus infection. Chin. Med. J..

[114] Zou X., Chen K., Zou J., Han P., Hao J., Han Z. (2020). Single-cell RNA-seq data analysis on the receptor ACE2 expression reveals the potential risk of different human organs vulnerable to 2019-nCoV infection. Front. Med..

[115] Kissling S., Rotman S., Gerber C., Halfon M., Lamoth F., Comte D., Lhopitallier L., Sadallah S., Fakhouri F. (2020). Collapsing glomerulopathy in a COVID-19 patient. Kidney Int..

[116] Varga Z., Flammer A.J., Steiger P., Haberecker M., Andermatt R., Zinkernagel A.S., Mehra M.R., Schuepbach R.A., Ruschitzka F., Moch H. (2020). Endothelial cell infection and endotheliitis in COVID-19. Lancet.

[117] Bradley B.T., Maioli H., Johnston R., Chaudhry I., Fink S.L., Xu H., Najafian B., Deutsch G., Lacy J.M., Williams T. (2020). Histopathology and ultrastructural findings of fatal COVID-19 infections in Washington State: A case series. Lancet.

[118] Pesaresi M., Pirani F., Tagliabracci A., Valsecchi M., Procopio A.D., Busardo F.P., Graciotti L. (2020). SARS-CoV-2 identification in lungs, heart and kidney specimens by transmission and scanning electron microscopy. Eur. Rev. Med. Pharmacol. Sci..

[119] Puelles V.G., Lutgehetmann M., Lindenmeyer M.T., Sperhake J.P., Wong M.N., Allweiss L., Chilla S., Heinemann A., Wanner N., Liu S. (2020). Multiorgan and Renal Tropism of SARS-CoV-2. N. Engl. J. Med..

[120] Braun F., Lutgehetmann M., Pfefferle S., Wong M.N., Carsten A., Lindenmeyer M.T., Norz D., Heinrich F., Meissner K., Wichmann D. (2020). SARS-CoV-2 renal tropism associates with acute kidney injury. Lancet.

[121] Santoriello D., Khairallah P., Bomback A.S., Xu K., Kudose S., Batal I., Barasch J., Radhakrishnan J., D’Agati V., Markowitz G. (2020). Postmortem Kidney Pathology Findings in Patients with COVID-19. J. Am. Soc. Nephrol..

[122] Dargelos M., Couturier A., Ferlicot S., Goujon J.M., Roque-Afonso A.M., Gault E., Touchard G., Ory C., Kaaki S., Vilaine E. (2020). Severe acute respiratory syndrome coronavirus 2 indirectly damages kidney structures. Clin. Kidney J..

[123] Best Rocha A., Stroberg E., Barton L.M., Duval E.J., Mukhopadhyay S., Yarid N., Caza T., Wilson J.D., Kenan D.J., Kuperman M. (2020). Detection of SARS-CoV-2 in formalin-fixed paraffin-embedded tissue sections using commercially available reagents. Lab. Investig..

[124] Delorey T.M., Ziegler C.G.K., Heimberg G., Normand R., Yang Y., Segerstolpe A., Abbondanza D., Fleming S.J., Subramanian A., Montoro D.T. (2021). COVID-19 tissue atlases reveal SARS-CoV-2 pathology and cellular targets. Nature.

[125] Dorward D.A., Russell C.D., Um I.H., Elshani M., Armstrong S.D., Penrice-Randal R., Millar T., Lerpiniere C.E.B., Tagliavini G., Hartley C.S. (2021). Tissue-Specific Immunopathology in Fatal COVID-19. Am. J. Respir. Crit. Care Med..

[126] Peng L., Liu J., Xu W., Luo Q., Chen D., Lei Z., Huang Z., Li X., Deng K., Lin B. (2020). SARS-CoV-2 can be detected in urine, blood, anal swabs, and oropharyngeal swabs specimens. J. Med. Virol..

[127] Sun J., Zhu A., Li H., Zheng K., Zhuang Z., Chen Z., Shi Y., Zhang Z., Chen S.B., Liu X. (2020). Isolation of infectious SARS-CoV-2 from urine of a COVID-19 patient. Emerg. Microbes Infect..

[128] Jeong H.W., Kim S.M., Kim H.S., Kim Y.I., Kim J.H., Cho J.Y., Kim S.H., Kang H., Kim S.G., Park S.J. (2020). Viable SARS-CoV-2 in various specimens from COVID-19 patients. Clin. Microbiol. Infect..

[129] Kim J.M., Kim H.M., Lee E.J., Jo H.J., Yoon Y., Lee N.J., Son J., Lee Y.J., Kim M.S., Lee Y.P. (2020). Detection and Isolation of SARS-CoV-2 in Serum, Urine, and Stool Specimens of COVID-19 Patients from the Republic of Korea. Osong Public Health Res. Perspect..

[130] Ling Y., Xu S.B., Lin Y.X., Tian D., Zhu Z.Q., Dai F.H., Wu F., Song Z.G., Huang W., Chen J. (2020). Persistence and clearance of viral RNA in 2019 novel coronavirus disease rehabilitation patients. Chin. Med. J..

[131] Zhang N., Gong Y., Meng F., Shi Y., Wang J., Mao P., Chuai X., Bi Y., Yang P., Wang F. (2021). Comparative study on virus shedding patterns in nasopharyngeal and fecal specimens of COVID-19 patients. Sci. China Life Sci..

[132] Zheng S., Fan J., Yu F., Feng B., Lou B., Zou Q., Xie G., Lin S., Wang R., Yang X. (2020). Viral load dynamics and disease severity in patients infected with SARS-CoV-2 in Zhejiang province, China, January-March 2020: Retrospective cohort study. BMJ.

[133] Wolfel R., Corman V.M., Guggemos W., Seilmaier M., Zange S., Muller M.A., Niemeyer D., Jones T.C., Vollmar P., Rothe C. (2020). Virological assessment of hospitalized patients with COVID-2019. Nature.

[134] Lescure F.X., Bouadma L., Nguyen D., Parisey M., Wicky P.H., Behillil S., Gaymard A., Bouscambert-Duchamp M., Donati F., Le Hingrat Q. (2020). Clinical and virological data of the first cases of COVID-19 in Europe: A case series. Lancet Infect. Dis..

[135] Mondanizadeh M., Hrahimi E., Sarmadian H., Jamalian M., Khansarinejad B. (2020). Evaluation of SARS-CoV-2 Existence in Blood, Urine, and Rectal Swab in Positive Patients with Different Virus Titers. Jundishapur J. Microbiol..

[136] To K.K., Tsang O.T., Leung W.S., Tam A.R., Wu T.C., Lung D.C., Yip C.C., Cai J.P., Chan J.M., Chik T.S. (2020). Temporal profiles of viral load in posterior oropharyngeal saliva samples and serum antibody responses during infection by SARS-CoV-2: An observational cohort study. Lancet Infect. Dis..

[137] Kujawski S.A., Wong K.K., Collins J.P., Epstein L., Killerby M., Midgley C.M., Abedi G.R., Ahmed N.S., Almendares O., Alvarez F.N. (2020). COVID-19 Investigation Team, Clinical and virologic characteristics of the first 12 patients with coronavirus disease 2019 (COVID-19) in the United States. Nat. Med..

[138] Chan J.F., Yip C.C., To K.K., Tang T.H., Wong S.C., Leung K.H., Fung A.Y., Ng A.C., Zou Z., Tsoi H.W. (2020). Improved Molecular Diagnosis of COVID-19 by the Novel, Highly Sensitive and Specific COVID-19-RdRp/Hel Real-Time Reverse Transcription-PCR Assay Validated In Vitro and with Clinical Specimens. J. Clin. Microbiol..

[139] Xie C., Jiang L., Huang G., Pu H., Gong B., Lin H., Ma S., Chen X., Long B., Si G. (2020). Comparison of different samples for 2019 novel coronavirus detection by nucleic acid amplification tests. Int. J. Infect. Dis..

[140] Trypsteen W., Van Cleemput J., Snippenberg W.V., Gerlo S., Vandekerckhove L. (2020). On the whereabouts of SARS-CoV-2 in the human body: A systematic review. PLoS Pathog..

[141] Bwire G.M., Majigo M.V., Njiro B.J., Mawazo A. (2021). Detection profile of SARS-CoV-2 using RT-PCR in different types of clinical specimens: A systematic review and meta-analysis. J. Med. Virol..

[142] De Souza N. (2018). Organoids. Nat. Methods.

[143] Buzhor E., Harari-Steinberg O., Omer D., Metsuyanim S., Jacob-Hirsch J., Noiman T., Dotan Z., Goldstein R.S., Dekel B. (2011). Kidney spheroids recapitulate tubular organoids leading to enhanced tubulogenic potency of human kidney-derived cells. Tissue Eng. Part A.

[144] Garreta E., Prado P., Tarantino C., Oria R., Fanlo L., Marti E., Zalvidea D., Trepat X., Roca-Cusachs P., Gavalda-Navarro A. (2019). Fine tuning the extracellular environment accelerates the derivation of kidney organoids from human pluripotent stem cells. Nat. Mater..

[145] Monteil V., Kwon H., Prado P., Hagelkruys A., Wimmer R.A., Stahl M., Leopoldi A., Garreta E., Hurtado Del Pozo C., Prosper F. (2020). Inhibition of SARS-CoV-2 Infections in Engineered Human Tissues Using Clinical-Grade Soluble Human ACE2. Cell.

[146] Hoffmann M., Kleine-Weber H., Schroeder S., Kruger N., Herrler T., Erichsen S., Schiergens T.S., Herrler G., Wu N.H., Nitsche A. (2020). SARS-CoV-2 Cell Entry Depends on ACE2 and TMPRSS2 and Is Blocked by a Clinically Proven Protease Inhibitor. Cell.

[147] Ogando N.S., Dalebout T.J., Zevenhoven-Dobbe J.C., Limpens R., van der Meer Y., Caly L., Druce J., de Vries J.J.C., Kikkert M., Barcena M. (2020). SARS-coronavirus-2 replication in Vero E6 cells: Replication kinetics, rapid adaptation and cytopathology. J. Gen. Virol..

[148] V’Kovski P., Kratzel A., Steiner S., Stalder H., Thiel V. (2021). Coronavirus biology and replication: Implications for SARS-CoV-2. Nat. Rev. Microbiol..

[149] Coutard B., Valle C., de Lamballerie X., Canard B., Seidah N.G., Decroly E. (2020). The spike glycoprotein of the new coronavirus 2019-nCoV contains a furin-like cleavage site absent in CoV of the same clade. Antiviral Res..

[150] Hasan A., Paray B.A., Hussain A., Qadir F.A., Attar F., Aziz F.M., Sharifi M., Derakhshankhah H., Rasti B., Mehrabi M. (2021). A review on the cleavage priming of the spike protein on coronavirus by angiotensin-converting enzyme-2 and furin. J. Biomol. Struct. Dyn..

[151] Yamada Y., Liu D.X. (2009). Proteolytic activation of the spike protein at a novel RRRR/S motif is implicated in furin-dependent entry, syncytium formation, and infectivity of coronavirus infectious bronchitis virus in cultured cells. J. Virol..

[152] Tchesnokov E.P., Feng J.Y., Porter D.P., Gotte M. (2019). Mechanism of Inhibition of Ebola Virus RNA-Dependent RNA Polymerase by Remdesivir. Viruses.

[153] Wang Y., Zhang D., Du G., Du R., Zhao J., Jin Y., Fu S., Gao L., Cheng Z., Lu Q. (2020). Remdesivir in adults with severe COVID-19: A randomised, double-blind, placebo-controlled, multicentre trial. Lancet.

[154] Beigel J.H., Tomashek K.M., Dodd L.E., Mehta A.K., Zingman B.S., Kalil A.C., Hohmann E., Chu H.Y., Luetkemeyer A., Kline S. (2020). Remdesivir for the Treatment of COVID-19—Final Report. N. Engl. J. Med..

[155] Heald-Sargent T., Gallagher T. (2012). Ready, set, fuse! The coronavirus spike protein and acquisition of fusion competence. Viruses.

[156] Soleimani M. (2020). Acute Kidney Injury in SARS-CoV-2 Infection: Direct Effect of Virus on Kidney Proximal Tubule Cells. Int. J. Mol. Sci..

[157] Wang M., Cao R., Zhang L., Yang X., Liu J., Xu M., Shi Z., Hu Z., Zhong W., Xiao G. (2020). Remdesivir and chloroquine effectively inhibit the recently emerged novel coronavirus (2019-nCoV) in vitro. Cell Res..

[158] Yao X., Ye F., Zhang M., Cui C., Huang B., Niu P., Liu X., Zhao L., Dong E., Song C. (2020). In Vitro Antiviral Activity and Projection of Optimized Dosing Design of Hydroxychloroquine for the Treatment of Severe Acute Respiratory Syndrome Coronavirus 2 (SARS-CoV-2). Clin. Infect. Dis..

[159] Shang C., Zhuang X., Zhang H., Li Y., Zhu Y., Lu J., Ge C., Cong J., Li T., Tian M. (2021). Inhibitors of endosomal acidification suppress SARS-CoV-2 replication and relieve viral pneumonia in hACE2 transgenic mice. Virol. J..

[160] Horby P., Mafham M., Linsell L., Bell J.L., Staplin N., Emberson J.R., Wiselka M., Ustianowski A., Elmahi E., RECOVERY Collaborative Group (2020). Effect of Hydroxychloroquine in Hospitalized Patients with COVID-19. N. Engl. J. Med..

[161] Tang W., Cao Z., Han M., Wang Z., Chen J., Sun W., Wu Y., Xiao W., Liu S., Chen E. (2020). Hydroxychloroquine in patients with mainly mild to moderate coronavirus disease 2019: Open label, randomised controlled trial. BMJ.

[162] Cavalcanti A.B., Zampieri F.G., Rosa R.G., Azevedo L.C.P., Veiga V.C., Avezum A., Damiani L.P., Marcadenti A., Kawano-Dourado L., Lisboa T. (2020). Hydroxychloroquine with or without Azithromycin in Mild-to-Moderate COVID-19. N. Engl. J. Med..

[163] Self W.H., Semler M.W., Leither L.M., Casey J.D., Angus D.C., Brower R.G., Chang S.Y., Collins S.P., Eppensteiner J.C., Filbin M.R. (2020). Effect of Hydroxychloroquine on Clinical Status at 14 Days in Hospitalized Patients with COVID-19: A Randomized Clinical Trial. JAMA.

[164] Arabi Y.M., Gordon A.C., Derde L.P.G., Nichol A.D., Murthy S., Beidh F.A., Annane D., Swaidan L.A., Beane A., Beasley R. (2021). Lopinavir-ritonavir and hydroxychloroquine for critically ill patients with COVID-19: REMAP-CAP randomized controlled trial. Intensive Care Med..

[165] Liu X., Liu X., Xu Y., Xu Z., Huang Y., Chen S., Li S., Liu D., Lin Z., Li Y. (2020). Ventilatory Ratio in Hypercapnic Mechanically Ventilated Patients with COVID-19-associated Acute Respiratory Distress Syndrome. Am. J. Respir. Crit. Care Med..

[166] Oppenheimer B.W., Bakker J., Goldring R.M., Teter K., Green D.L., Berger K.I. (2020). Increased Dead Space Ventilation and Refractory Hypercapnia in Patients with Coronavirus Disease 2019: A Potential Marker of Thrombosis in the Pulmonary Vasculature. Crit. Care Explor..

[167] South A.M., Tomlinson L., Edmonston D., Hiremath S., Sparks M.A. (2020). Controversies of renin-angiotensin system inhibition during the COVID-19 pandemic. Nat. Rev. Nephrol..

[168] Jiang X., Eales J.M., Scannali D., Nazgiewicz A., Prestes P., Maier M., Denniff M., Xu X., Saluja S., Cano-Gamez E. (2020). Hypertension and renin-angiotensin system blockers are not associated with expression of angiotensin-converting enzyme 2 (ACE2) in the kidney. Eur. Heart J..

[169] Milne S., Yang C.X., Timens W., Bosse Y., Sin D.D. (2020). SARS-CoV-2 receptor ACE2 gene expression and RAAS inhibitors. Lancet Respir. Med..

[170] Wysocki J., Lores E., Ye M., Soler M.J., Batlle D. (2020). Kidney and Lung ACE2 Expression after an ACE Inhibitor or an Ang II Receptor Blocker: Implications for COVID-19. J. Am. Soc. Nephrol..

[171] Mancia G., Rea F., Ludergnani M., Apolone G., Corrao G. (2020). Renin-Angiotensin-Aldosterone System Blockers and the Risk of COVID-19. N. Engl. J. Med..

[172] Morales D.R., Conover M.M., You S.C., Pratt N., Kostka K., Duarte-Salles T., Fernandez-Bertolin S., Aragon M., DuVall S.L., Lynch K. (2021). Renin-angiotensin system blockers and susceptibility to COVID-19: An international, open science, cohort analysis. Lancet Digit. Health.

[173] Reynolds H.R., Adhikari S., Pulgarin C., Troxel A.B., Iturrate E., Johnson S.B., Hausvater A., Newman J.D., Berger J.S., Bangalore S. (2020). Renin-Angiotensin-Aldosterone System Inhibitors and Risk of COVID-19. N. Engl. J. Med..

[174] Zhang P., Zhu L., Cai J., Lei F., Qin J.J., Xie J., Liu Y.M., Zhao Y.C., Huang X., Lin L. (2020). Association of Inpatient Use of Angiotensin-Converting Enzyme Inhibitors and Angiotensin II Receptor Blockers with Mortality among Patients with Hypertension Hospitalized with COVID-19. Circ. Res..

[175] Baral R., Tsampasian V., Debski M., Moran B., Garg P., Clark A., Vassiliou V.S. (2021). Association Between Renin-Angiotensin-Aldosterone System Inhibitors and Clinical Outcomes in Patients with COVID-19: A Systematic Review and Meta-analysis. JAMA Netw. Open.

[176] Bavishi C., Whelton P.K., Mancia G., Corrao G., Messerli F.H. (2021). Renin-angiotensin-system inhibitors and all-cause mortality in patients with COVID-19: A systematic review and meta-analysis of observational studies. J. Hypertens..

[177] Pranata R., Permana H., Huang I., Lim M.A., Soetedjo N.N.M., Supriyadi R., Soeroto A.Y., Alkatiri A.A., Firman D., Lukito A.A. (2020). The use of renin angiotensin system inhibitor on mortality in patients with coronavirus disease 2019 (COVID-19): A systematic review and meta-analysis. Diabetes Metab. Syndr..

[178] Cohen J.B., Hanff T.C., William P., Sweitzer N., Rosado-Santander N.R., Medina C., Rodriguez-Mori J.E., Renna N., Chang T.I., Corrales-Medina V. (2021). Continuation versus discontinuation of renin-angiotensin system inhibitors in patients admitted to hospital with COVID-19: A prospective, randomised, open-label trial. Lancet Respir. Med..

[179] Lopes R.D., Macedo A.V.S., de Barros E.S.P.G.M., Moll-Bernardes R.J., Dos Santos T.M., Mazza L., Feldman A., D’Andrea Saba Arruda G., de Albuquerque D.C., Camiletti A.S. (2021). Effect of Discontinuing vs Continuing Angiotensin-Converting Enzyme Inhibitors and Angiotensin II Receptor Blockers on Days Alive and Out of the Hospital in Patients Admitted with COVID-19: A Randomized Clinical Trial. JAMA.

[180] Duarte M., Pelorosso F., Nicolosi L.N., Salgado M.V., Vetulli H., Aquieri A., Azzato F., Castro M., Coyle J., Davolos I. (2021). Telmisartan for treatment of COVID-19 patients: An open multicenter randomized clinical trial. EClinicalMedicine.

[181] Touyz R.M., Montezano A.C. (2018). Angiotensin-(1-7) and Vascular Function: The Clinical Context. Hypertension.

[182] Liu M., Wang T., Zhou Y., Zhao Y., Zhang Y., Li J. (2020). Potential Role of ACE2 in Coronavirus Disease 2019 (COVID-19) Prevention and Management. J. Transl. Int. Med..

[183] Wosten-van Asperen R.M., Lutter R., Specht P.A., Moll G.N., van Woensel J.B., van der Loos C.M., van Goor H., Kamilic J., Florquin S., Bos A.P. (2011). Acute respiratory distress syndrome leads to reduced ratio of ACE/ACE2 activities and is prevented by angiotensin-(1-7) or an angiotensin II receptor antagonist. J. Pathol..

[184] Imai Y., Kuba K., Rao S., Huan Y., Guo F., Guan B., Yang P., Sarao R., Wada T., Leong-Poi H. (2005). Angiotensin-converting enzyme 2 protects from severe acute lung failure. Nature.

[185] Zou Z., Yan Y., Shu Y., Gao R., Sun Y., Li X., Ju X., Liang Z., Liu Q., Zhao Y. (2014). Angiotensin-converting enzyme 2 protects from lethal avian influenza A H5N1 infections. Nat. Commun..

[186] Gu H., Xie Z., Li T., Zhang S., Lai C., Zhu P., Wang K., Han L., Duan Y., Zhao Z. (2016). Angiotensin-converting enzyme 2 inhibits lung injury induced by respiratory syncytial virus. Sci. Rep..

[187] Khan A., Benthin C., Zeno B., Albertson T.E., Boyd J., Christie J.D., Hall R., Poirier G., Ronco J.J., Tidswell M. (2017). A pilot clinical trial of recombinant human angiotensin-converting enzyme 2 in acute respiratory distress syndrome. Crit. Care.

[188] Ozkan S., Cakmak F., Konukoglu D., Biberoglu S., Ipekci A., Akdeniz Y.S., Bolayirli I.M., Balkan I.I., Dumanli G.Y., Ikizceli I. (2021). Efficacy of Serum Angiotensin II Levels in Prognosis of Patients with Coronavirus Disease 2019. Crit. Care Med..

[189] Wang K., Gheblawi M., Nikhanj A., Munan M., MacIntyre E., O’Neil C., Poglitsch M., Colombo D., Del Nonno F., Kassiri Z. (2022). Dysregulation of ACE (Angiotensin-Converting Enzyme)-2 and Renin-Angiotensin Peptides in SARS-CoV-2 Mediated Mortality and End-Organ Injuries. Hypertension.

[190] Haga S., Yamamoto N., Nakai-Murakami C., Osawa Y., Tokunaga K., Sata T., Yamamoto N., Sasazuki T., Ishizaka Y. (2008). Modulation of TNF-alpha-converting enzyme by the spike protein of SARS-CoV and ACE2 induces TNF-alpha production and facilitates viral entry. Proc. Natl Acad Sci. USA.

[191] Palau V., Riera M., Soler M.J. (2020). ADAM17 inhibition may exert a protective effect on COVID-19. Nephrol. Dial. Transplant..

[192] Dudoignon E., Moreno N., Deniau B., Coutrot M., Longer R., Amiot Q., Mebazaa A., Pirracchio R., Depret F., Legrand M. (2020). Activation of the renin-angiotensin-aldosterone system is associated with Acute Kidney Injury in COVID-19. Anaesth. Crit. Care Pain Med..

[193] Jayk Bernal A., Gomes da Silva M.M., Musungaie D.B., Kovalchuk E., Gonzalez A., Delos Reyes V., Martin-Quiros A., Caraco Y., Williams-Diaz A., Brown M.L. (2021). Molnupiravir for Oral Treatment of COVID-19 in Nonhospitalized Patients. N. Engl. J. Med..

[194] Ng S., Cowling B.J., Fang V.J., Chan K.H., Ip D.K., Cheng C.K., Uyeki T.M., Houck P.M., Malik Peiris J.S., Leung G.M. (2010). Effects of oseltamivir treatment on duration of clinical illness and viral shedding and household transmission of influenza virus. Clin. Infect. Dis..

[195] Siddiqi H.K., Mehra M.R. (2020). COVID-19 illness in native and immunosuppressed states: A clinical-therapeutic staging proposal. J. Heart Lung Transplant..

[196] Sinha P., Matthay M.A., Calfee C.S. (2020). Is a “Cytokine Storm” Relevant to COVID-19?. JAMA Intern. Med..

[197] Kox M., Waalders N.J.B., Kooistra E.J., Gerretsen J., Pickkers P. (2020). Cytokine Levels in Critically Ill Patients with COVID-19 and Other Conditions. JAMA.

[198] Leisman D.E., Ronner L., Pinotti R., Taylor M.D., Sinha P., Calfee C.S., Hirayama A.V., Mastroiani F., Turtle C.J., Harhay M.O. (2020). Cytokine elevation in severe and critical COVID-19: A rapid systematic review, meta-analysis, and comparison with other inflammatory syndromes. Lancet Respir. Med..

[199] Mudd P.A., Crawford J.C., Turner J.S., Souquette A., Reynolds D., Bender D., Bosanquet J.P., Anand N.J., Striker D.A., Martin R.S. (2020). Distinct inflammatory profiles distinguish COVID-19 from influenza with limited contributions from cytokine storm. Sci. Adv..

[200] Horby P., Lim W.S., Emberson J.R., Mafham M., Bell J.L., Linsell L., Staplin N., Brightling C., Ustianowski A., RECOVERY Collaborative Group (2021). Dexamethasone in Hospitalized Patients with COVID-19. N. Engl. J. Med..

[201] Sterne J.A.C., Murthy S., Diaz J.V., Slutsky A.S., Villar J., Angus D.C., Annane D., Azevedo L.C.P., Berwanger O., WHO Rapid Evidence Appraisal for COVID-19 Therapies (REACT) Working Group (2020). Association Between Administration of Systemic Corticosteroids and Mortality among Critically Ill Patients with COVID-19: A Meta-analysis. JAMA.

[202] Gordon A.C., Mouncey P.R., Al-Beidh F., Rowan K.M., Nichol A.D., Arabi Y.M., Annane D., Beane A., van Bentum-Puijk W., REMAP-CAP Investigators (2021). Interleukin-6 Receptor Antagonists in Critically Ill Patients with COVID-19. N. Engl. J. Med..

[203] RECOVERY Collaborative Group (2021). Tocilizumab in patients admitted to hospital with COVID-19 (RECOVERY): A randomised, controlled, open-label, platform trial. Lancet.

[204] Marconi V.C., Ramanan A.V., de Bono S., Kartman C.E., Krishnan V., Liao R., Piruzeli M.L.B., Goldman J.D., Alatorre-Alexander J., de Cassia Pellegrini R. (2021). Efficacy and safety of baricitinib for the treatment of hospitalised adults with COVID-19 (COV-BARRIER): A randomised, double-blind, parallel-group, placebo-controlled phase 3 trial. Lancet Respir. Med..

[205] Katzourakis A. (2022). COVID-19: Endemic doesn’t mean harmless. Nature.

